# Correlation analysis of the transcriptome of growing leaves with mature leaf parameters in a maize RIL population

**DOI:** 10.1186/s13059-015-0735-9

**Published:** 2015-09-11

**Authors:** Joke Baute, Dorota Herman, Frederik Coppens, Jolien De Block, Bram Slabbinck, Matteo Dell’Acqua, Mario Enrico Pè, Steven Maere, Hilde Nelissen, Dirk Inzé

**Affiliations:** Department of Plant Systems Biology, Vlaams Instituut voor Biotechnologie, Technologiepark 927, 9052 Ghent, Belgium; Department of Plant Biotechnology and Bioinformatics, Ghent University, Technologiepark 927, 9052 Ghent, Belgium; Institute of Life Sciences, Scuola Superiore Sant’Anna, Piazza Martiri della Libertà 33, 56127 Pisa, Italy

## Abstract

**Background:**

To sustain the global requirements for food and renewable resources, unraveling the molecular networks underlying plant growth is becoming pivotal. Although several approaches to identify genes and networks involved in final organ size have been proven successful, our understanding remains fragmentary.

**Results:**

Here, we assessed variation in 103 lines of the *Zea mays* B73xH99 RIL population for a set of final leaf size and whole shoot traits at the seedling stage, complemented with measurements capturing growth dynamics, and cellular measurements. Most traits correlated well with the size of the division zone, implying that the molecular basis of final leaf size is already defined in dividing cells of growing leaves. Therefore, we searched for association between the transcriptional variation in dividing cells of the growing leaf and final leaf size and seedling biomass, allowing us to identify genes and processes correlated with the specific traits. A number of these genes have a known function in leaf development. Additionally, we illustrated that two independent mechanisms contribute to final leaf size, maximal growth rate and the duration of growth.

**Conclusions:**

Untangling complex traits such as leaf size by applying in-depth phenotyping allows us to define the relative contributions of the components and their mutual associations, facilitating dissection of the biological processes and regulatory networks underneath.

**Electronic supplementary material:**

The online version of this article (doi:10.1186/s13059-015-0735-9) contains supplementary material, which is available to authorized users.

## Background

Leaves are the main organs for photosynthesis of the plant and thus have an indispensable role in the generation of metabolic energy and organic compounds [1]. The typical laminar and flat morphology of leaves is ideally suited to capture light energy during photosynthesis. Leaf size is an important component of plant architecture that determines in part the amount of energy that can be captured, and as such has a profound effect on productivity. Therefore, understanding the molecular mechanisms underlying plant leaf growth and final size is a major goal for plant science. The monocotyledonous plant *Zea mays* (maize) shows a high level of intraspecific phenotypic variation, making it excellently suited for genomic approaches and to study complex phenotypes such as leaf size. Furthermore, the large size of the maize leaf makes it easier to dissect specific organ domains [2, 3].

At the cellular level, leaf size is determined by two processes, cell proliferation and cell expansion, which are highly coordinated [3]. In maize, growing leaves show a developmental gradient from base to tip, making the maize leaf an interesting model to study growth [3, 4]. The different phases of maize leaf development have been described in detail [5]. In the first phase after emergence of the leaf primordium from the shoot apical meristem, cell division and expansion take place simultaneously so that the mean cell size remains constant and the complete leaf consists of dividing cells in the so-called division zone (DZ). In the next phase, cells stop dividing at the tip of the leaf, but continue to expand post-mitotically, giving rise to the expansion zone, distal to the DZ. In the third phase, cells enter the mature zone at the tip of the leaf where cells stop expanding. In this phase of development, the leaf appears from the sheath of the surrounding older leaves and shows a developmental gradient from base to tip, with dividing, expanding and mature cells [3, 4]. Also, the size of the DZ remains constant during this phase as well as the elongation rate of the leaf [5]. Finally, the elongation rate decreases due to a regression of the growth zone.

Although leaf development is well described at the cellular level, knowledge on the molecular mechanisms that determine leaf growth and final size is still fragmentary, due to the complex polygenic control of these traits (e.g., [3, 6–12]). Several approaches have been followed to dissect the genetic circuits that underlie leaf growth. Forward genetics screens have proven to be useful in the identification of genes that have the potential to contribute to natural variation of phenotypic traits and their specific function [13]. However, the majority of the mutant screens have been limited to a small number of laboratory strains, which harbor only a small portion of the natural variation [14]. Therefore, exploring natural variation provides a complementary resource to identify novel genes and allelic variants, especially for quantitative traits [15]. Typically, linkage analysis is performed using recombinant inbred line (RIL) populations to identify genomic regions with at least one gene controlling part of the phenotypic variability (e.g., [16–19]). Alternatively, genome-wide association analysis in natural populations identifies causative single nucleotide polymorphisms (SNPs) for specific traits [20]. However, these approaches are time consuming and the regions identified by linkage mapping and the SNPs detected using genome-wide association analysis often contain a large number of candidate genes that need to be further narrowed down using complementary analyses or a priori knowledge [21, 22].

A complementary approach that became available thanks to the recent development of new “-omics” tools is high throughput profiling of large mapping populations, offering new perspectives for genetic integration of several levels of molecular regulation of phenotypic trait variation [23]. In maize, differences in gene expression patterns are suggested to be a more important cause of subtle changes in quantitative traits than alterations in protein sequences causing defective proteins [24–27]. Therefore, exploring transcriptome variation in mapping populations has great potential to characterize the regulatory mechanisms and candidate genes that are at the basis of phenotypic differences [28].

Gene expression analyses in growing tissues have revealed that the transition from dividing and expanding to mature tissue coincides with vast transcriptional changes and differences in protein levels [2, 29–32]. In the leaf basal region, transcripts and proteins related to primary cellular metabolism, such as DNA/RNA-related processes, cell growth and regulation/signaling, are more abundant, transitioning to enrichment in transcripts and proteins for secondary cell wall biosynthesis and photosynthetic development towards the mature region of the leaf. Thus, to study the molecular mechanisms underlying leaf growth, it is important to focus on the growth zone. More specifically, since the majority of the growth regulatory genes that have been described so far affect the final number of cells rather than the final size of the cells [3, 10, 33], focusing on transcriptional changes in the DZ is expected to have the largest potential for finding new regulatory genes for final leaf size.

The use of new transcriptomics tools has resulted in the generation of large amounts of tissue-specific expression data that have led to new insights into the molecular basis of leaf development [2, 30]. However, the number of studies that link differences in expression levels to phenotypic measurements on a large scale remains limited up to now [34–36]. On the other hand, approaches such as genome-wide association and linkage mapping typically use measurements at the whole-organ or organismal scale, resulting in information that is often too complex to dissect out the biological processes and regulatory interactions involved [37]. In this study we combined a detailed phenotypic analysis of maize seedlings, focusing on leaf size, with transcript profiling of dividing leaf tissue of the B73xH99 recombinant inbred line population [38] to further unravel the molecular basis of leaf development. Phenotyping included a set of final leaf measurements, i.e., leaf length, leaf width, leaf area and leaf weight, and whole-shoot measurements at the seedling stage, i.e., fresh weight, dry weight, leaf number and V-stage. These end-point measurements were combined with measurements that capture growth dynamics, i.e., maximal growth rate of the leaf and traits related to timing of leaf development. The latter concern emergence of the fourth leaf above the pseudostem cylinder made by sheaths of previously emerged leaves and duration of elongation. Finally, leaf development was partially assessed at the cellular level by determining the size of the DZ during steady state growth of the fourth leaf in all RILs. Capturing dynamic and cellular measurements could reveal new regulatory genes related to more specific processes compared with only considering end point measurements [37, 39]. Correlation analysis between these phenotypic traits revealed that the size of the DZ is positively correlated with most of the final size traits, supporting the hypothesis that the molecular basis underlying final leaf size is already determined in dividing cells of a growing leaf. To further decipher these molecular networks, we captured the transcriptional differences in dividing cells early during leaf development in a RIL population and combined this with the detailed phenotyping. Several genes and processes were identified that show an association between phenotype and expression levels in dividing cells in this RIL population, and that have orthologs in other species with known leaf size phenotypes upon perturbation. We focused on some specific associations between the rate and duration of elongation and final organ size and the association between maximal growth rate and seedling biomass. The identification of candidate genes — novel genes as well as known growth regulators — not only ameliorates our knowledge of the gene network underlying leaf development but also provides a framework to identify transcriptional markers for breeding new varieties and offers opportunities for genetic modification approaches.

## Results and discussion

### The size of the DZ correlates with final leaf size, shoot and growth parameters

We phenotyped an established F12 RIL population derived from the inbred parents B73 and H99 [40] for leaf- and shoot-related traits at the seedling stage (Table 1; Fig. S1 in Additional file 1). The majority of the traits are linked to the final size of the fourth leaf: final leaf length (LL), final leaf width (Lwi), final leaf area (LA) and final leaf weight (Lwe). In addition, we determined the size of the DZ and leaf elongation rate (LER) — the estimation of the growth of an individual leaf in a given time frame — during the steady state growth phase [41] (see "Material and methods"). To complement final size measurements and further capture leaf growth dynamics, we measured some timing-related traits for leaf 4 using LEAF-E [42]: (i) time point of emergence of leaf 4 from the whorl; (ii) time between sowing and reaching final length (T_e_); (iii) time between sowing and reaching maximal growth rate (T_m_); and (iv) leaf elongation duration from a leaf of 5 mm until final length (LED_5-e_). Together these traits provide an estimate of how long the plant needs to fully expand leaf 4. In addition to these leaf size-related traits, we determined fresh weight (FW) and dry weight (DW) of the above soil-grown plant parts at 27 days after sowing, i.e., when leaf 4 had reached its final size in all lines, and we counted leaf number (LN) and V-stage at this time point.

**Table 1:** Mean, maximum, minimum and percentage differences of the traits determined for the 103 RILs

Trait	Mean ± SD	Maximum	Minimum	Percentage difference	B73	H99	*P* value
Final leaf length (mm)	623.0 ± 63.6	789.0	469.0	41	624.9 ± 8.5	584.5 ± 6.7	<0.05
Final leaf weight (g)	4.69 ± 1.05	7.82	2.56	67	4.17 ± 0.13	4.32 ± 0.12	NS
Final leaf area (mm^2^)	80.39 ± 15.09	122.71	46.82	62	74.07 ± 2.30	69.81 ± 1.53	NS
Final leaf width (mm)	2.57 ± 0.31	3.43	1.61	53	2.34 ± 0.04	2.48 ± 0.03	<0.05
Leaf elongation rate (mm/h)	2.73 ± 0.27	3.46	2.07	40	2.93 ± 0.04	2.41 ± 0.03	<0.05
Emergence of leaf 4 (days)	12.44 ± 0.87	14.80	10.35	30	11.40 ± 0.07	12.18 ± 0.14	<0.05
T_e_ (h)	510.5 ± 38.4	618.5	435.9	30	462.2 ± 3.7	519.7 ± 5.2	<0.05
T_m_ (h)	405.8 ± 33.9	499.3	336.3	33	363.0 ± 2.9	409.1 ± 4.4	<0.05
LED5-e (h)	369.7 ± 27.6	443.0	307.4	31	341.9 ± 3.5	381.3 ± 3.7	<0.05
Division zone size (μm)	11,082 ± 1,790	16,800	7,817	53	10,908 ± 312	11,650 ± 1,176	NS
Number of leaves at harvest	8.4 ± 0.6	9.8	7.2	27	9.2 ± 0.1	8.0 ± 0.1	<0.05
V-stage at harvest	5.0 ± 0.4	6.0	4.0	33	5.5 ± 0.1	4.9 ± 0.1	<0.05
Shoot fresh weight (g)	33.3 ± 7.5	49.9	19.5	61	40.6 ± 1.1	31.5 ± 1.2	<0.05
Shoot dry weight (g)	2.6 ± 0.6	4.4	1.7	63	3.5 ± 0.1	2.2 ± 0.1	<0.05

Measurements are averages of 18–20 plants per RIL for leaf 4 emergence, of three plants per RIL for division zone size, and of six plants per RIL for all other traits. *P* values are for differences between B73 and H99 parents. *LED*_*5-e*_ leaf elongation duration from a leaf of 5 mm until final length, *NS* not significant, *T*_*e*_ time between sowing and reaching final length, *T*_*m*_ time between sowing and reaching maximal growth rate

For the majority of the traits, the two parental lines clearly differed and encompassed the RIL average values (Table 1; Fig. S1 in Additional file 1), but the average of the two parents did not differ significantly from the average over all RILs (*p* > 0.05). In general, B73 plants were larger than H99 at the seedling stage: FW and DW were higher for B73 than for H99 (*p* < 0.05), which was in part due to the larger number of leaves (and corresponding V-stage; *p* < 0.05) (Fig. S2 in Additional file 1). In agreement, timing traits showed that B73 leaves developed faster (emergence, T_e_, T_m_ and LED_5-e_ are smaller, *p* < 0.05). The fourth leaf of B73 grew faster during steady state and was longer and larger when fully grown than that of H99, but was somewhat narrower (*p* < 0.05). Nonetheless, DZ size was not significantly different between B73 and H99 (*p* > 0.05). In previous reports on the B73-H99 RIL population, traits were measured for fully grown plants, e.g., flowering and yield components, and a trend was also observed for higher values for B73 than for H99 [38, 43, 44].

Screening 103 RIL lines showed that the weight-related parameters (FW, DW and Lwe) displayed the largest variation (about 65 %), while the smallest variation (about 30 %) was seen for the timing parameters emergence, T_m_, T_e_ and LED_5-e_ (Table 1; Fig. S3 in Additional file 1).

Next, Pearson correlation coefficients (PCCs) were determined between all traits obtained for all RILs and the parental lines (Table 2). Traits related to final leaf size (Lwe, LA, LL and Lwi) correlated well, and the shoot traits (DW, FW, LN and V-stage) also showed a positive correlation, as did the timing traits (emergence, T_m_, T_e_ and LED_5-e_). DZ size correlated positively with the final leaf size traits and LER. Also, DZ size correlated to some extent with FW and DW, while no significant correlation between DZ size and timing traits was observed. LER correlated positively with most of the final leaf size traits and with FW and DW, but with none of the timing traits. We also performed a principal component analysis (PCA) to validate the relationships between the phenotypic traits; we focused on the first two principal components, which explain most variance within the data (Fig. S4 in Additional file 1). Based on PCA and correlation analysis, phenotypic traits were separated into three groups (Table 2; Fig. S4 in Additional file 1): leaf size traits (final size traits LL, Lwe, LA and Lwi, and in addition LER and DZ size), shoot traits (FW, DW, LN and V-stage) and timing traits (emergence, T_m_, T_e_ and LED_5-e_).

**Table 2:** Pearson correlation coefficients for the different traits analyzed

		Leaf size traits						Timing traits				Shoot traits			
		LL	Lwe	LA	Lwi	LER	DZ size	Emergence	Te	Tm	LED_5-e_	LN	V-stage	FW	DW
Leaf size traits	LL	1	0.750**	0.781**	0.316**	0.738**	0.594**	0.245*	0.379**	0.374**	0.424**	−0.255	−0.423**	0.215*	0.206*
	Lwe		1	0.915**	0.688**	0.493**	0.516**	0.258**	0.328**	0.334**	0.332**	−0.258	−0.420**	0.413**	0.311**
	LA			1	0.794**	0.479**	0.526**	0.291**	0.414**	0.413**	0.418**	−0.273	−0.472**	0.297**	0.245*
	Lwi				1	0.063	0.308**	0.307**	0.365**	0.379**	0.298**	−0.267	−0.375*	0.170	0.098
	LER					1	0.507**	0.007	−0.105	−0.048	−0.114	0.176	0.083	0.520**	0.502**
	DZ size						1	0.097	0.113	0.118	0.147	−0.251	−0.266	0.211*	0.219*
Timing traits	Emergence							1	0.620**	0.650**	0.461**	−0.649**	−0.599**	−0.455**	−0.462**
	T_e_								1	0.979**	0.894**	−0.621**	−0.701**	−0.582**	−0.557**
	T_m_									1	0.787**	−0.601**	−0.675**	−0.568**	−0.557**
	LED_5-e_										1	−0.568**	−0.656**	−0.470**	−0.420**
Shoot traits	LN											1	0.834**	0.521**	0.377*
	V-stage												1	0.435**	0.326*
	FW													1	0.893**
	DW														1

Significant correlations are indicated by **p < 0.01 and *p < 0.05; highly significant positive correlations are indicated in bold; highly significant negative correlations are indicated in italics. *LL* leaf length, *Lwe* leaf weight, *LA* leaf area, *Lwi* leaf width, *LER* leaf 4 elongation rate, *DZ* division zone, *T*_*m*_ time to maximal LER, *T*_*e*_ time to final leaf length, *LED*_*5-e*_ leaf elongation duration, *FW* shoot fresh weight, *DW* shoot dry weight, *LN* leaf number

The leaf size traits Lwe and LER correlated well with the shoot traits FW and DW. In agreement, co-localization of quantitative trait loci (QTL) for leaf growth rate and growth of other organs suggests that the growth rates in different organs share a part of their genetic control [16], implying that similar genes and networks of genes affect organ growth and, as a consequence, also biomass accumulation. In wheat and tall fescue, leaf area expansion rate also correlated positively with above-ground biomass and grain yield [45, 46]. V-stage showed a negative correlation with the final leaf size traits, suggesting that plants with larger leaves were generally slower in producing new leaves. Accordingly, timing traits showed a positive correlation with traits related to final leaf size, implying that larger leaves needed more time to obtain their final size compared with smaller leaves. In agreement, there was a negative correlation between whole shoot traits and timing traits, also illustrated in the biplot of the PCA (Fig. S4 in Additional file 1). This implies that if it takes longer for a plant to obtain its final leaf size, this generally results in fewer leaves and smaller shoot biomass when leaf 4 stops growing.

The positive correlation between DZ size and the final leaf size parameters confirmed the importance of the number of dividing cells in determination of final organ size. Previously, it was shown that cell proliferation, and more specifically the transition between cell division and cell expansion, is an important contributing factor to final organ size in different plant species [3, 47, 48]. Additionally, perturbation of most leaf size regulatory genes primarily affects cell number rather than cell size [3, 33, 49, 50]. This suggests that focusing on transcriptional differences between RILs in the DZ may provide deeper insight into the molecular networks behind final leaf size traits. Since the DZ size varied considerably between RILs (Table 1) and we wanted to avoid sampling expanding tissue, we restricted our analysis to the most basal 0.5 cm of the DZ, which contained only proliferating cells in all analyzed RILs.

### Transcriptome analysis of proliferative tissue confirms the correlation between leaf size, growth and shoot parameters

We performed RNA sequencing to profile transcriptional changes in proliferative leaf tissue of 103 lines of the B73xH99 RIL population. To this end, the most basal 0.5 cm of the fourth leaf was sampled during the steady state growth phase, i.e., three days after the tip of the fourth leaf emerged from the pseudostem cylinder. The B73 maize reference genome [51] was used to align the RNA sequencing data. As variations on the sequence level can affect the alignment of reads to the reference genome, differences in the genetic background of the two parental lines could affect gene expression quantification differently in different RILs. Therefore, we focused on conserved genes, selecting expressed genes with a low percentage of SNPs across maize inbred lines (see "Material and methods"). This resulted in a filtered set of 15,051 genes that were used for all further analyses.

**Fig. 1:**
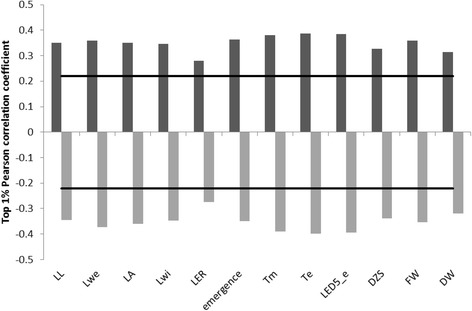
Correlation coefficients of the top 1 % (anti-)correlating genes. The q_0.99_ and q_0.01_ quantiles of the distributions of PCCs between transcript expression levels and phenotypes, for real data (q_0.99_ in *dark grey* and q_0.01_ quantile in *light grey*) and permuted data (*black line*). *LL* leaf 4 final length, *Lwe* leaf 4 final weight, *LA* leaf 4 final area, *Lwi* leaf 4 final width, *LER* leaf 4 elongation rate, *T*_*m*_ time to maximal LER, *T*_*e*_ time to final leaf length, *LED*_*5-e*_ leaf elongation duration, *DZS* leaf 4 DZ size, *FW* fresh weight 27 days after sowing, *DW* dry weight 27 days after sowing

The expression data and the phenotypic data were combined after normalization by calculating PCCs between transcript expression values and trait values across all RILs. From the probability plots, the q_0.99_ and q_0.01_ correlation coefficients were determined, i.e., the 1 % best correlating and anti-correlating transcripts. The q_0.99_ and q_0.01_ correlation coefficients were also determined after permutation of the phenotypic data over the RILs (Fig. 1), revealing that we could identify genes correlated higher than expected with a certain phenotype compared with a random gene set. The number of genes correlating better than random, indicated further as the q_random_ correlating gene sets, varied between 1073 and 3276 depending on the phenotype (Table 3). The maximal correlation coefficients were rather low, ranging from 0.456 for DZ size to 0.617 for T_e_, which is not unexpected since the traits under study are polygenic [6, 19, 52] and known to be controlled by a large number of small-effect genes [53]. For all traits, the numbers of positively and negatively correlating genes in the q_random_ correlating gene sets were similar. The numbers of genes in the q_random_ correlating gene sets for FW and DW were comparable to the numbers found for leaf size traits, which was unexpected since DW and FW are believed to be more complex traits than the others. Although we cannot exclude that additional and/or partially different diagnostic transcriptional variation would be captured in the complete DZ, our results imply that the transcriptome of the most basal part of the DZ during steady state growth of a leaf at least partly reflects final organ size and even more distant phenotypic traits such as FW and DW.

**Table 3:** Number of genes that correlate with a certain phenotypic trait higher than random

Phenotype	Correlation	Percentage	Positive correlation	Negative correlation
Leaf length	2206	15	1162	1044
Leaf weight	2596	18	1240	1356
Leaf area	2230	16	1084	1146
Leaf width	2477	17	1222	1255
Leaf elongation rate	1073	8	498	575
Emergence	2490	17	1337	1153
T_m_	3171	22	1730	1441
T_e_	3276	23	1748	1528
LED_5-e_	3003	21	1563	1440
Division zone size	1927	14	881	1046
Fresh weight	2259	16	994	1256
Dry weight	1707	12	746	961

**Fig. 2:**
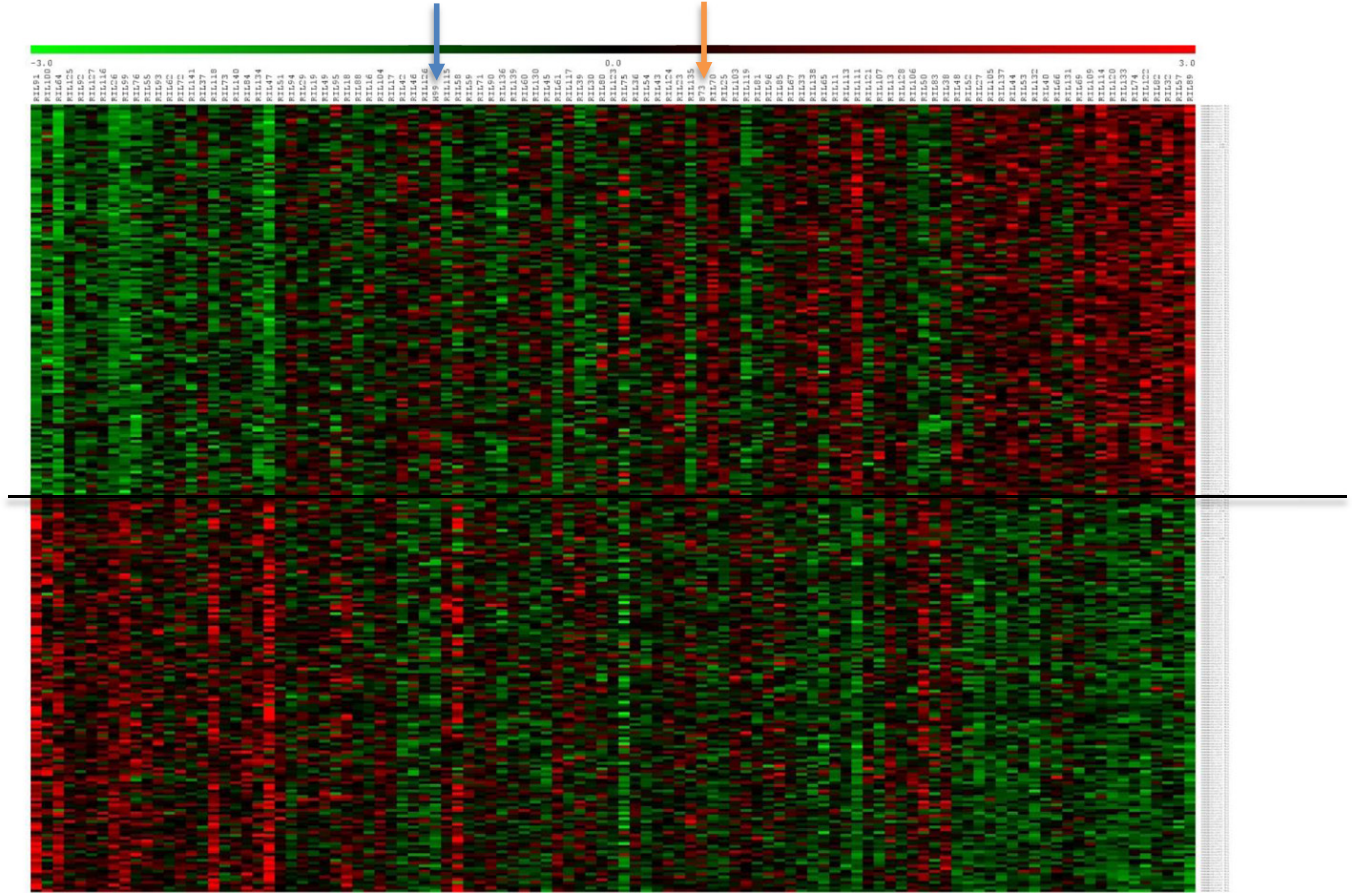
Expression patterns of the top 1 % genes (anti-)correlated with leaf length. Columns represent the 103 RILs and parental lines B73 and H99, organized from small (*left*) to large (*right*) leaf length; rows represent gene expression profiles. Genes above the line are significantly correlated with leaf length; genes below the line are significantly anti-correlated with leaf length. Parental lines H99 and B73 are indicated by the *blue arrow* and *orange arrow*, respectively. *Green* indicates low expression, *red* indicates high expression

For the remaining analyses, we focused on the 1 % best correlating and anti-correlating genes for each of the traits, further indicated as the correlated and anti-correlated gene sets. These gene sets are for each trait subsets of the q_random_ correlated gene sets discussed above (Fig. 1). Visualization of the expression pattern of these anti-correlating/correlating genes in the 103 RILs and the parents is exemplified in Fig. 2 for leaf length (and for other traits in Fig. S5 in Additional file 1). When the RILs were ordered according to phenotype from smallest to largest RIL, a clear correlation with gene expression was observed. The observed gradient became, as expected, less clear for the traits for which the correlation coefficients were lower, such as for LER and DW (Fig. S5 in Additional file 1). Although we could identify individual genes for which expression level is clearly associated with one or more of the traits we analyzed, the low correlation levels and the observation that no single gene was for all RILs associated with a particular trait across all RILs suggests that not one gene but a network of multiple genes is underlying the traits under study.

**Fig. 3:**
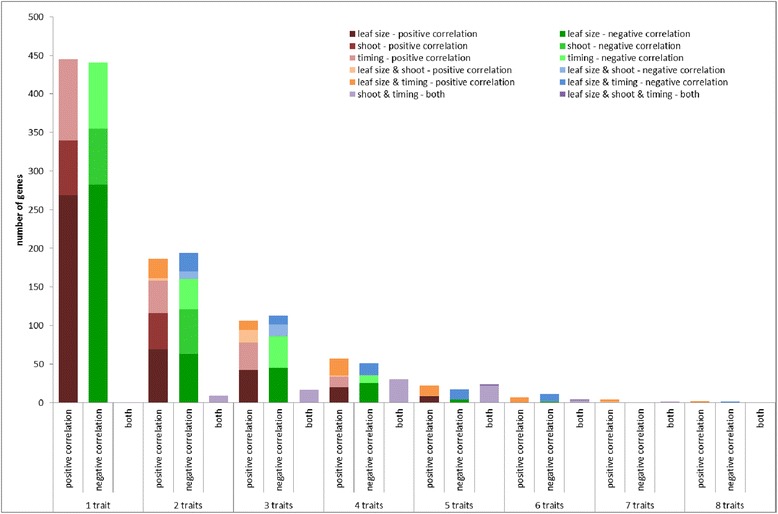
Number of genes correlating or anti-correlating with one or multiple traits. Traits were separated into three groups: leaf size traits (leaf elongation rate, leaf length, leaf weight, leaf area, leaf width and DZ size), shoot traits (fresh weight and dry weight) and timing traits (emergence, T_m_, T_e_ and leaf elongation duration). Positive correlation is colored *red* (traits of the same group) and *orange* (traits of different groups), negative correlation is colored *green* (traits of the same group) and *blue* (traits of different groups), while *purple bars* indicate genes that show positive correlation with one trait and negative correlation with another trait

In total, 1740 genes are part of the (anti-)correlating gene sets of at least one of the traits (Additional file 2). Approximately half of these genes — 886 genes or 51 % — were specific for one trait, while three genes (anti-)correlated with eight traits, the maximum number of traits for which there were genes in common (Fig. 3). These genes have no immediate known link with leaf development (*GRMZM2G166713*, which shows homology to a methionine tRNA synthetase; *GRMZM2G471142*, which shows homology to barley MLO genes; and a third gene, *GRMZM2G389768*, which shows homology to cold shock domain-containing proteins). The numbers of genes (anti-)correlating with from one to eight traits were comparable for positive and negative correlation. Comparing the numbers of genes (anti-)correlating with two or more traits within and between the three phenotype classes defined based on their correlation (leaf size, timing and shoot traits) showed that this number is higher within groups than between groups (this is illustrated in Fig. 3; compare the size of the red bars versus the orange bars and the green bars versus the blue bars). The number of common genes between leaf size traits and shoot traits was limited — just 50 genes. Furthermore, only a minority of the genes — 84 genes or 5 % — correlated with one or more traits and anti-correlated with one or more other traits (purple bars in Fig. 3). This opposite correlation for different traits was exclusively observed for timing traits versus shoot traits; strikingly, there was not one (anti)-correlating gene in common between shoot and timing traits. Thus, the (anti-)correlation between traits on a phenotypic level was fully supported by the correlations between expression levels of the selected genes and traits.

In a next step we evaluated if the correlated genes for the different traits were enriched in comparable processes (Fig. S6 in Additional file 1). Enrichment for specific processes was calculated based on MapMan gene function annotations [54]. For positively correlated gene sets, there was an enrichment in six categories: “regulation of transcription”, “hormone metabolism”, “protein modifications”, “protein degradation” “carbohydrate metabolism” and “transport”. Negatively correlated gene sets were enriched for the categories “regulation of transcription”, “cell wall synthesis and degradation” and “protein synthesis”. Most of the enriched categories were not specific for one trait, with the exception of the category “transport” for genes correlating with leaf emergence, although no specificity for transport of certain compounds was found. Furthermore, we found that traits were enriched for the same processes not only when they have a large number of correlating genes in common (e.g., FW and DW), but also when only a limited number of genes was shared (e.g., the shoot traits and final leaf size traits). Functional categories “carbohydrate metabolism” and “transport” were specific for the timing related traits.

**Fig. 4:**
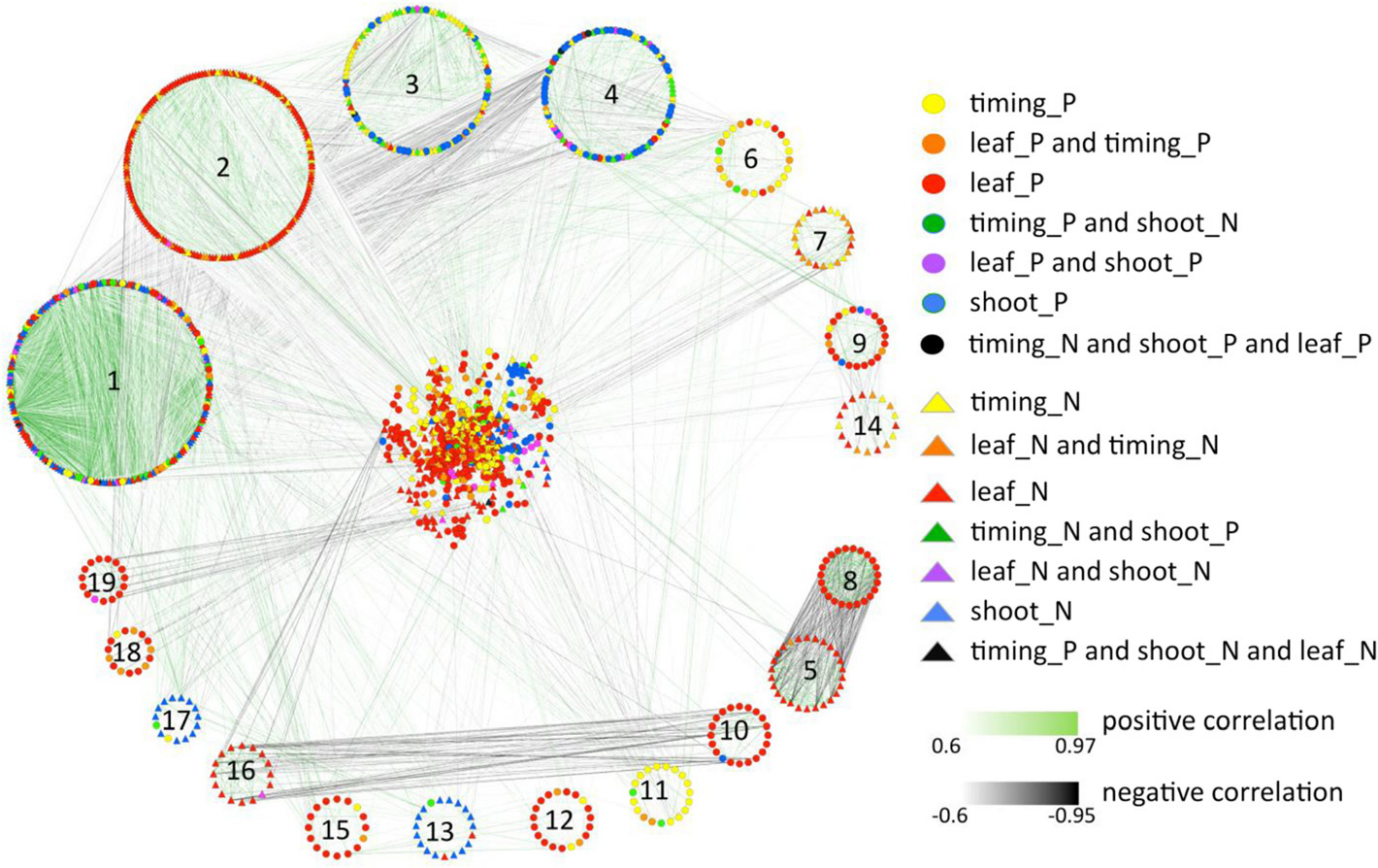
Transcript co-expression network based on Pearson correlation. The network of transcript co-expression links with correlation coefficients higher than 0.6 or lower than −0.6 as visualized in Cytoscape [148]. A circular layout is used for 19 clusters that contain at least 15 nodes. The remaining transcripts are displayed in the middle with a prefuse force directed layout. Node colors and shapes represent the associations of transcripts with the traits (in the figure legend, *_P* stands for positive and *_N* for negative correlation with the trait concerned), whereby similarly colored *circles* and *triangles* depict opposite associations with the same traits. The strength of the correlations between transcripts is reflected in the hue of the edges

To visualize the co-expression of the genes correlating with the traits, we generated a correlation network starting from the 1740 genes that (anti-)correlated to at least one of the traits. The network was based on correlation coefficients between the transcripts (nodes) higher than 0.6 or lower than −0.6, and as such 1459 transcripts were connected by 23,363 edges. The network was clustered using the Markov cluster algorithm (MCL) [55] (see "Materials and methods" for details). The algorithm differentiated 155 clusters encompassing all but 81 transcripts (Fig. 4). For 19 clusters containing more than 15 nodes, we calculated enrichment for specific processes in a comparable way as for the trait-specific gene sets [54]. Eight of the 19 clusters were significantly enriched for one or more functional categories (clusters 1, 2, 3, 4, 6, 7, 9, and 14). Cluster 1 was enriched for protein-related processes, i.e., “amino acid activation” and “protein degradation and protein targeting”, and for “cell vesicle transport”, although the genes in this cluster were not associated with any specific trait (Fig. 4). Cluster 2, with mainly genes anti-correlated to leaf size and timing traits, was found to be enriched for the functional categories “protein synthesis”, “regulation of transcription”, “photosynthesis” and “tetrapyrrole synthesis” (Fig. 4). Clusters 3 and 4 consisted primarily of nodes positively correlating with shoot traits or negatively correlating with timing traits. Cluster 3 was enriched for functional categories “protein synthesis” and “DNA synthesis/chromatin structure”. Cluster 4 was found to be enriched in “hormone metabolism” and “regulation of transcription”, both functional categories positively correlated with shoot traits, in accordance with the predominantly shoot trait-correlating gene content of this cluster (Fig. 4). Cluster 6, representing predominantly genes correlated with timing traits, was enriched in the functional category “major carbohydrate metabolism” (Fig. 4). Clusters 7 and 14, containing nodes anti-correlated with leaf size and timing traits, were enriched in the category “cell wall degradation and synthesis” (Fig. 4). Cluster 9, positively correlated with leaf size traits, was enriched in “regulation of transcription” (Fig. 4).

Taken together, some of the gene expression-based clusters associated with certain traits or combination of traits contain genes assigned to functional categories found in the overall enrichment results. Most of the gene clusters and most of the categories found enriched in those clusters are not specific for one trait group, but for a combination. This is not unexpected given the correlations we find between the traits on the phenotype level. Also, some functional categories were found enriched in several clusters associated with uncorrelated trait groups, or inversely correlated with a particular trait group, e.g., “regulation of transcription” in clusters 2 (negatively correlated with leaf size traits), 4 (positively correlated with shoot traits) and 9 (positively correlated with leaf size traits).

### Phenotypic components and genes contributing to final leaf size

To decipher the complexity of final leaf biomass, this trait was dissected in different components. In general, correlation analysis showed significant positive correlations between the different components, consisting of leaf length, leaf weight, leaf area and leaf width (LL, Lwe, LA and Lwi), although the strength of the relationships differed considerably (Table 2). The PCC between LA and Lwe was high (0.915), implying that the contribution of leaf thickness to Lwe is negligible. This is in agreement with currently used models for leaf and crop growth, where leaf thickness is not taken into consideration [56]. Comparable PCCs between LL–LA/Lwe and Lwi–LA/Lwe (0.781/0.750 and 0.794/0.688, respectively), while correlation between Lwi and LL was only limited (0.316), suggest an equal contribution of LL and Lwi to final LA and Lwe. Comparably, the overlap between LL and Lwi at the genetic level in the nested association mapping population in maize was very restricted [18], and meta-analysis of several populations also confirmed a low correlation coefficient between LL and Lwi [57].

**Fig. 5:**
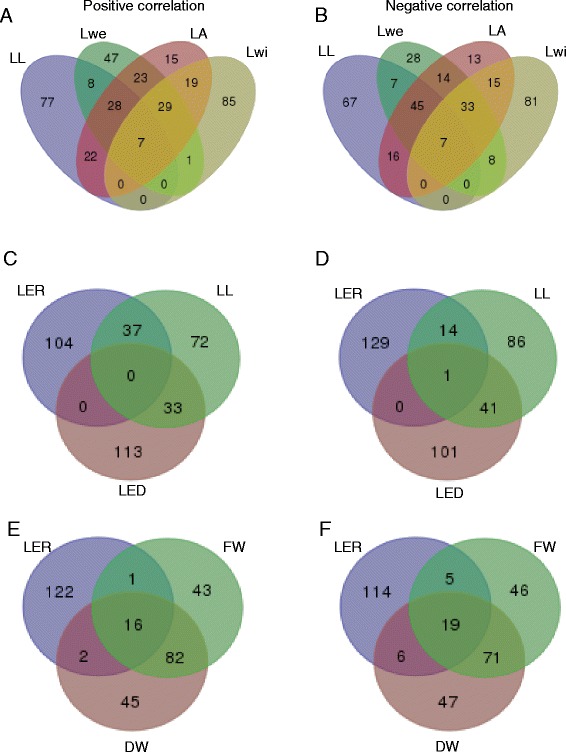
Venn diagrams of the top 1% transcripts that correlate positively or negatively with selected phenotypic traits. **a** Transcripts positively correlated with final leaf size traits (leaf length, leaf weight, leaf area and leaf width). **b** Transcripts negatively correlated with final leaf size traits (leaf length, leaf weight, leaf area and leaf width). **c** Transcripts positively correlated with leaf elongation rate, leaf elongation duration and leaf length. **d** Transcripts negatively correlated with leaf elongation rate, leaf elongation duration and leaf length. **e** Transcripts positively correlated with fresh weight, dry weight and leaf elongation rate. **f** Transcripts negatively correlated with fresh weight, dry weight and leaf elongation rate

The diversity in associations between the final leaf size traits was also reflected in the transcriptome data. Figure 5a and b represent the intersections between the genes correlating and anti-correlating, respectively, with the final leaf size traits. Of the in total 361 and 334 genes correlating and anti-correlating, respectively, with at least one of the final leaf size traits, 224 and 189 were specific for one particular trait. Lwi in particular only shared a limited number of genes with the other traits, suggesting that this trait is under different genetic control than the other final leaf size traits.

Consistent with the co-expression network and enrichment analysis for separate traits (Fig. 4; Fig. S6 in Additional file 1), the genes positively correlating with the final leaf size traits were enriched for four functional categories of genes: “regulation of transcription”, “protein degradation”, “protein modifications” and “hormone metabolism”. Negatively correlating transcripts were enriched for the categories “cell wall synthesis and degradation”, “protein synthesis”, “tetrapyrrole synthesis”, and “photosynthesis” (Fig. S7 in Additional file 1). The biological significance of the major categories of positively and negatively correlating transcripts is discussed below and examples of (orthologous) genes with a known function in leaf development are summarized in Table 4.

**Table 4:** Examples of genes for which expression levels are (anti-)correlated with leaf size-related traits

MapMan	Gene	Maize description	*Arabidopsis* orthologs	*Arabidopsis* symbol	*Arabidopsis* description	LL	Lwe	LA	Lwi	*Arabidopsis* orthologs with phenotype	Phenotype	Reference
Regulation of transcription	*GRMZM2G361659*		AT3G48160^a^	DEL1, E2FE, E2L3	DP-E2F-like 1	−−	−	−		AT3G48160^a^	Overexpression results in smaller leaves due to repression of cell proliferation	[76]
			AT3G01330^c^									
			AT5G14960^c^									
	*GRMZM2G462623*		AT5G02470^b^ AT5G03415^a^	DPA, DPB	Transcription factor DP	++	++	++		AT5G02470^b^	DPa acts together with E2F as stimulator of cell proliferation	[75]
	*GRMZM2G099862*	Putative growth-regulating factor	AT2G22840^b^	ATGRF1, ATGRF2	Growth-regulating factor 1, growth-regulating factor 2	++	+	+		AT2G22840^b^	Overexpression results in larger cotyledons and leaves due to increase in cell number	[74]
			AT4G37740^a,b^							AT4G37740^a,b^		
	*GRMZM2G470307*		AT4G32730^b^	ATMYB3R-1, ATMYB3R1, MYB3R-1, MYB3R1, PC-MYB1, AtMYB3R4, MYB3R-4	Homeodomain-like protein, myb domain protein 3r-4		+	+	++	AT5G11510^a,b^	Loss of function mutants are more compact	[77]
			AT5G11510^a,b^									
	*GRMZM2G053298*		AT1G20640^b^		Plant regulator RWP-RK family protein	+	++	++	+	AT1G20640^b^	Loss of function mutants have smaller rosettes	[77]
			AT1G76350^a,b,c^									
			AT2G17150^b,c^									
			AT4G35270^b^									
			AT4G38340^b^									
	*GRMZM2G067624*		AT1G53160^a,b^	SPL4, FTM6, SPL3, SPL5	Squamosa promoter binding protein-like, FLORAL TRANSITION AT THE MERISTEM6	+	++	++	+	AT1G53160^a,b^	Promotor of vegetative phase change; influencing the duration of the vegetative phase can affect number and size of leaves	[78, 149]
			AT2G33810^b^									
			AT3G15270^b^									
Hormone metabolism	*GRMZM2G144701*		AT2G42820^c^	HVA22F	HVA22-like protein F	+	++	+		AT2G42820^c^	Downregulation of HVA22-like genes results in plants with dwarf and bushy stature with a reduced seed set	[80]
	*GRMZM2G130548*	HVA22-like protein a	AT1G74520^a^	HVA22A	HVA22 homologue A		++	++	++	AT1G74520^a^	Downregulation of HVA22-like genes results in plants with dwarf and bushy stature with a reduced seed set	[80]
	*GRMZM2G102347*		AT1G75700^a,b^	HVA22G	HVA22-like protein G	++				AT1G75700^a,b^	Downregulation of HVA22-like genes results in plants with dwarf and bushy stature with a reduced seed set	[80]
			AT5G42560^b^									
			AT1G19950^b^									
	*GRMZM2G135978*		AT1G12820^c^	AFB3, AFB2, TIR1, AFB1, GRH1	Auxin signaling F-box 3, auxin signaling F-box 2, F-box/RNI-like superfamily protein, GRR1-like protein 1	++	+	+		AT3G62980^a,b,c^	Mutations in TIR1 that enhance the degradation of auxin/IAA display more lateral roots, smaller rosettes and reduced number of axillary branches	[59, 77]
			AT3G26810^c^									
			AT3G62980^a,b,c^									
			AT4G03190^b,c^									
	*GRMZM2G057000*	Brassinosteroid biosynthesis-like protein	AT3G19820^a,b,c^	CBB1, DIM, DIM1, DWF1, EVE1	Cell elongation protein/DWARF1/DIMINUTO (DIM)	−−	−	−		AT3G19820^a,b,c^	Brassinosteroid dwarfs have more roundish, dark green leaves next to short, robust inflorescence and reduced fertility	[81]
	*GRMZM2G125943*	Histidine kinase	AT1G27320^c^	AHK3, HK3, AHK4, ATCRE1, CRE1, WOL, WOL1, AHK2, HK2	Histidine kinase 3, CHASE domain containing histidine kinase protein, histidine kinase 2	+	++	++	+	AT1G27320^c^	Ectopic expression of ZmHK6 in *Arabidopsis* results in strongly enhanced shoot development, next to smaller seeds and a smaller root system; loss of function mutants in *Arabidopsis* orthologs have severe phenotypes, including smaller leaves due to a strong reduction in the number of cells	[150, 151]
			AT2G01830^a,b^							AT2G01830^a,b^		
			AT5G35750^c^							AT5G35750^c^		
	*GRMZM2G158252*	Histidine kinase 3	AT1G27320^c^ AT5G35750^a,b,c^	AHK3, HK3, AHK4, ATCRE1, CRE1, WOL, WOL1, AHK2, HK2	Histidine kinase 3, CHASE domain containing histidine kinase protein, histidine kinase 2	++	++	++	+	AT1G27320^c^ AT5G35750^a,b,c^	Ectopic expression of ZmHK1 in *Arabidopsis* results in strongly enhanced shoot development, next to smaller seeds and a smaller root system; loss of function mutants in *Arabidopsis* orthologs have severe phenotypes, including smaller leaves due to a strong reduction in the number of cells	[150, 151]
	*GRMZM2G067225*	Allene oxide synthase	AT5G42650^a,b,c^	DDE2, AOS, CYP74A	DELAYED DEHISCENCE 2, allene oxide synthase, CYTOCHROME P450 74A	−−	−	−	−	AT5G42650^a,b,c^	aos mutants have larger leaves and rosettes	[83, 152]
Protein degradation	*GRMZM2G041561*	BRCA1-associated protein	AT2G26000^a,b,c^	BRIZ2	Zinc finger (C3HC4-type RING finger) family protein	++	++	++	++	AT2G26000^a,b,c^	Loss of function mutants show severe phenotype; genes involved in seed germination and early seedling growth	[91]
	*GRMZM2G120408*		AT3G61590^a,b^	HWS, HS	HAWAIIAN SKIRT	++	+	+		AT3G61590^a,b^	Loss of function mutants display increased growth of leaves and roots, while overexpression reduces rosette size	[92]
	*GRMZM2G116314*		AT1G17110^a,b,c^	UBP15	Ubiquitin-specific protease 15	+	++	+		AT1G17110^a,b,c^	Overexpression results in plants with larger rosettes due to increase in leaf weight and number of leaves, while mutants display opposite phenotypes	[90]
Posttranslational modifications	*GRMZM2G054634*	ATP binding protein	AT1G01740^c^	BSK3, BSK1, BSK2	BR-signaling kinase	++	+	++		AT1G01740^c^	Triple, quadruple and pentuple loss of function mutants show a reduced rosette size	[153]
			AT1G50990^c^							AT1G50990^c^		
			AT1G63500^c^							AT1G63500^c^		
			AT3G54030^c^							AT3G54030^c^		
			AT4G00710^c^							AT4G00710^c^		
			AT4G35230^c^							AT4G35230^c^		
			AT5G41260^c^							AT5G41260^c^		
			AT5G46570^a,b,c^							AT5G46570^a,b,c^		
			AT5G59010^c^							AT5G59010^c^		
	*GRMZM2G004572*		AT1G53730^a^	SRF6, SRF7, SRF4, SRF5, SRF3	STRUBBELIG-receptor family protein	+	++	++	+	AT3G13065^c^	Loss of function mutants show smaller leaves while overexpressing plants display enlarged leaves	[95]
			AT3G13065^c^									
			AT1G78980^c^									
			AT4G03390^c^									
Cell wall	*GRMZM2G328500*		AT1G26570^b,c^	UGD1, UGD2, UGD3	UDP-glucose dehydrogenase 1, UDP-glucose 6-dehydrogenase family protein	−−	−	−		AT3G29360^a,b,c^	Double mutants in the two isoforms of the enzyme udg2,3 are severely dwarfed due to defects in cell wall composition	[101]
			AT3G29360^a,b,c^							AT5G15490^b,c^		
			AT5G15490^b,c^									
			AT5G39320^b,c^									

*Arabidopsis* orthologs were determined using PLAZA3.0 web resource [147]. ^a^ Best hit family ortholog. ^b^ Tree-based ortholog. ^c^ Orthologous gene family. Double plus signs ("*++*") indicate transcript levels positively correlated with phenotypic trait (q_0.99_). Single plus signs ("*+*") indicate transcript levels positively correlated with phenotypic trait (q_random_). Double minus signs ("*−−*") indicate transcript levels negatively correlated with phenotypic trait (q_0.01_). Single minus signs ("*−*") indicate transcript levels negatively correlated with phenotypic trait (q_random_). *IAA* indole-3-acetic acid

Of the 15,051 genes in the filtered gene set, 1433 genes are part of the MapMan category “regulation of transcription”, and for 82 of these genes expression levels in the RILs were positively or negatively correlated with at least one of the final leaf size traits. These 82 genes were separated over 32 different families of transcription factors, although no clear trends of specificity of certain families for certain traits could be observed (Additional file 2). The *Arabidopsis* homologs of several of these genes are involved in hormone regulation, such as ARFs and AUX/IAA in auxin signaling [58, 59], ARR in cytokinin signaling [60] and GRAS in gibberellin signaling [61]. Some genes are homologs of the Alfin-like family, SET-domain family and DNA methyltransferases, all involved in regulation of chromatin structure in *Arabidopsis* [62–67], which is essential for normal cell functioning, development and leaf growth [50]. Other genes belong to (super)families of transcription factors that are functionally very diverse, e.g., the B3 superfamily [68], bHLH family [69], bZIP family [70], MYB family [71], NAC family [72] and Trihelix family [73]. Many of these families contain transcription factors with a clear role in leaf development; some examples are summarized in Table 4. For instance, *GRMZM2G099862* shows homology to the *Arabidopsis* transcriptional activators *GROWTH REGULATING FACTOR 1* (*GRF1*) and *GRF2* [74]; two E2F/DP transcription factors, *GRMZM2G462623* and *GRMZM2G361659*, showed an opposed correlation with leaf size traits, in agreement with the function of their putative homologs in *Arabidopsis* [75, 76]; *GRMZM2G470307* shows homology to *Arabidopsis* MYB domain protein 3r-4 [77]; *GRMZM2G053298* encodes a transcription factor with homology to an *Arabidopsis* plant regulator RWP-RK family protein [77]; *GRMZM2G067624* encodes a squamosa promoter binding protein with homology to *Arabidopsis* SPL4, which promotes vegetative phase change [78].

A second functional category, next to “regulation of transcription”, in which gene sets positively correlate with several of the final leaf size traits was enriched in “hormone regulation” (Fig. S7 in Additional file 1). Plant hormones regulate diverse processes in plant development and are suggested to play an important role in the balance between cell division and differentiation to modulate growth [79]. Sixteen genes encoding proteins for auxin, cytokinin, brassinosteroid, ethylene, abscisic acid, gibberellin and jasmonate biosynthesis and signaling correlated with final leaf size traits (Table 4; Additional file 2). Some of these genes have homologs in *Arabidopsis* which are known to function in leaf development and/or display defects in leaf morphology when perturbed and some examples are summarized in Table 4. For instance, *GRMZM2G135978* shows homology to the family of *Arabidopsis* TIR1/AFB auxin receptors [59]; *GRMZM2G130548*, *GRMZM2G144701* and *GRMZM2G102347* are putative *HVA22*-like genes [80]; *GRMZM2G057000* is the maize ortholog of *Arabidopsis DWARF1*, a brassinosteroid biosynthetic enzyme [81]; histidine kinase receptors (HK) ZmHK1 (*GRMZM2G158252*) and ZmHK6 (*GRMZM2G125943*) result in enhanced shoot development when ectopically expressed in *Arabidopsis* [82]; *GRMZM2G067225* is a homolog of the *Arabidopsis* jasmonate biosynthesis gene *ALLENE OXIDE SYNTHASE* (*AOS*) [83].

The functional category “protein degradation” was enriched in all gene sets positively correlated with a final leaf size trait (Fig. S6 in Additional file 1). Controlled proteolysis is an important layer of regulation, next to (post-)transcriptional and (post-)translational regulation. For instance, progression through the cell cycle requires tight control of the involved regulatory proteins and depends on a precise temporal and spatial proteolysis of these proteins through the ubiquitin-mediated pathway, next to other regulatory mechanisms such as phosphorylation/dephosphorylation and specific protein–protein interactions [84, 85]. Several genes that are part of the ubiquitin-mediated pathway display an altered final leaf size when perturbed, e.g., *APC10*, *SAMBA*, *DA1* and *BIG BROTHER* [86–89]. *GRMZM2G116314* is a maize homolog of *Arabidopsis UBIQUITIN*-*SPECIFIC PROTEASE 15* (*UBP15*), involved in protein de-ubiquitination [90]; *GRMZM2G041561* encodes a RING finger domain E3 ligase with homology to *Arabidopsis BRAP2 RING ZNF UBP DOMAIN-CONTAINING PROTEIN 2* (*BRIZ2*), essential for seed germination and for post-germination growth [91]; *GRMZM2G120408* is a maize homolog of the *Arabidopsis* F-box E3 ligase *HAWAIIAN SKIRT* (*HWS*) [92] (Table 4).

Besides the positive correlation with “protein degradation”, Lwe and LA were also positively correlated with genes involved in “protein modifications”, including posttranslational modifications and glycosylation. Because these modifications are essential to rapidly transduce inter- and intracellular information, they are important for regulation of plant growth and development [93]. *GRMZM2G054634* shows homology to *Arabidopsis BR*-*SIGNALING KINASES* (*BSK*) [94]; *GRMZM2G004572* is related to the STRUBBELIG-receptor family (SRF) in *Arabidopsis* [95] (Table 4).

The category “protein synthesis”, and more specifically synthesis of ribosomal proteins, was overrepresented in the anti-correlating gene sets for all final leaf size traits (Fig. 4; Fig. S6 in Additional file 1). Protein synthesis is one of the most energy consuming processes in the cell and a major component of cell growth. An indirect cost of protein synthesis is to synthesize and maintain the structural constituents of the ribosomes, the ribosomal proteins. A more efficient translational machinery can minimize these indirect costs and result in more energy available for growth [96–98], which might explain the anti-correlation we observed between ribosomal protein synthesis transcript levels and leaf size and timing traits. The fact that the translation machinery also plays a role in leaf development is supported by the many mutations in ribosomal proteins that have been reported to affect leaf morphology (reviewed in [99, 100]).

Genes negatively correlating with leaf size traits were also enriched for the functional category “cell wall synthesis and degradation” (Fig. 4; Fig. S6 in Additional file 1). Cell wall expansion is essential to allow cell growth and is thus pivotal for plant growth and development. *GRMZM2G328500* is a homolog of the *Arabidopsis* gene encoding UDP-glucose dehydrogenase 2 (UDG2), which is a key enzyme in primary cell wall formation [101] (Table 4).

The multitude of genes with expression levels correlating with final leaf size traits in this RIL population that have homologs in *Arabidopsis* showing altered leaf sizes upon perturbation implies that these genes are part of molecular networks in the most basal part of the DZ that are associated with final leaf size traits. For some genes (e.g., *GRMZM2G120408* and *GRMZM2G120408*), correlation in our dataset does not reflect the anticipated variation based on phenotypes of the *Arabidopsis* homologs. This is possibly due to comparing subtle variation in expression and allelic effects and combination of these within the RIL population with more abrupt effects of complete knock-out or strong overexpression, often in one or a limited number of genetic backgrounds in *Arabidopsis*.

### Both maximal growth rate and duration of growth independently determine final leaf length

The final length of a monocot leaf depends on the rate and duration of elongation of the leaf, represented here by the parameters LER and LED_5-e_. LER and LED_5-e_ showed no correlation on the phenotype level (Table 2), in contrast to the positive correlation between final size-related traits and timing traits on the one hand and LER on the other hand. A comparable correlation between LER and LL was reported for the sixth leaf in greenhouse and field conditions, and was supported by the co-location of QTL for LER and LL in several populations [16]. For *Lolium perenne* grown in the field, correlation between LER and LED was limited, while there was a high positive correlation between LER and LL, and between LED and LL. LER and LED were also not correlated in wheat (*Triticum aestivum*) and its wild relative *Aegilops* [102].

**Fig. 6:**
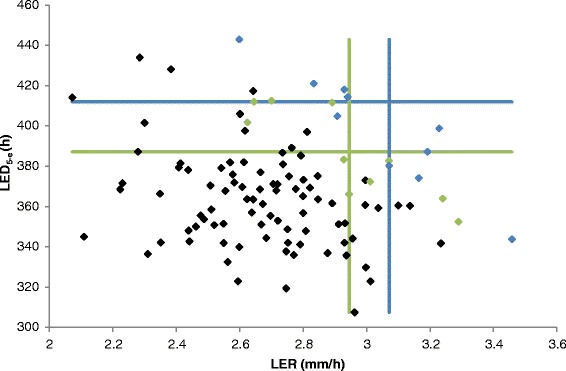
Scatter plot of the measurements for the leaf elongation rate (LER) and leaf elongation duration (LED_5-e_) traits. *Blue dots* indicate the 10 % of RILs with the largest leaf length; *green dots* the 20 % of RILs with largest leaf length. *Blue lines* represent the borders of the 10 % of RILs with largest LED_5-e_ and LER; *green lines* represent the borders of the 20 % of RILs with largest LED_5-e_ and LER

Figure 6 represents a scatter plot of LER and LED_5-e_, with the blue and green lines indicating the borders of the 10 % and 20 % largest RILs for the two traits, respectively. This figure illustrates that there is only a very limited number of lines that have both a high LER and LED_5-e_, supporting an idea of phenotypic tradeoff. Thus, a better insight into these two traits on the molecular level can provide useful information for selecting or engineering plants with increased biomass.

The fact that there is no correlation on the phenotype level between LER and LED_5-e_ is also reflected on the molecular level: no (anti-)correlating genes are shared between LER and LED_5-e_ (Fig. 5c, d), pointing to separate, independent molecular mechanisms that determine final leaf length. Given the high PCC (0.738) between LER and LL on the phenotype level, the number of genes specifically correlating with one of the traits was rather high — more than two-thirds of the LER-correlating genes were specific for LER (Table 2; Fig. 5c, d). Despite the lower PCC between LED_5-e_ and LL than between LER and LL, this was not reflected in the number of genes in these intersections (Fig. 5c, d). This is possibly due to the lower values for the 0.99 and 0.01 quantiles of Pearson correlation distributions for LER than for the other traits (Fig. 1). The limited overlap in (anti-)correlating genes between LL and LER or LED_5-e_ suggests that the molecular networks underlying elongation rate, elongation duration and final size are only partially shared and other additional mechanisms are also possibly involved.

On the gene level, the intersection between LER and LL represents genes functioning in hormone signal transduction and metabolism (auxin, brassinosteroid and ethylene), protein degradation machinery (E3 RING proteins), transcription factors (bZIP and E2F/DP) and genes related to transport, calcium and light signaling (Additional file 2). Some of these genes have *Arabidopsis* homologs that are possibly involved in regulation of leaf development, given that perturbation results in altered leaf and rosette sizes (Table 5). Examples are GRMZM2G462623 and GRMZM2G361659, two E2F/DP transcription factors showing homology to DPa and DEL1, respectively [75, 76], GRMZM2G135978, a putative ortholog of TRANSPORT INHIBITOR RESPONSE 1 (TIR1) [59, 77], and GRMZM2G445905, a cellulose synthase showing homology to IRREGULAR XYLEM 5 (IRX5) [103].

**Table 5:** Examples of genes for which expression levels are (anti-)correlated with leaf length, LER or LED_5-e_

MapMan	Gene	Maize description	*Arabidopsis* orthologs	*Arabidopsis* symbol	*Arabidopsis* description	LER	LL	LED_5_e_	*Arabidopsis* orthologs with phenotype	Phenotype	Reference
Regulation of transcription	*GRMZM2G702026*	Auxin response factor 1	AT1G59750^a,b^	ARF1	Auxin response factor 1		−−	−−	AT1G59750^a,b^	*arf1* mutations enhance *arf2* phenotype, i.e., delayed leaf senescence resulting in more and larger leaves	[77, 154]
	*GRMZM2G361659*		AT3G48160^a^	DEL1, E2FE, E2L3	DP-E2F-like 1	−−	−−		AT3G48160^a^	Overexpression results in smaller leaves due to repression of cell proliferation	[76]
			AT3G01330^b^								
			AT5G14960^b^								
	*GRMZM2G462623*		AT5G02470^b^ AT5G03415^a^	DPA, DPB	Transcription factor DP	++	++	+	AT5G02470^b^	DPa acts together with E2F as stimulator of cell proliferation	[75]
Hormone metabolism	*GRMZM2G135978*		AT1G12820^c^	AFB3, AFB2, TIR1, AFB1, GRH1	Auxin signaling F-box 3, auxin signaling F-box 2, F-box/RNI-like superfamily protein, GRR1-like protein 1	++	++		AT3G62980^a,b,c^	Mutations in TIR1 that enhance the degradation of auxin/IAA display more lateral roots, smaller rosettes and reduced number of axillary branches	[59, 77]
			AT3G26810^c^								
			AT3G62980^a,b,c^								
			AT4G03190^b,c^								
	*GRMZM2G074267*	Putative auxin efflux carrier	AT1G23080^c^	PIN7, PIN3, PIN1, PIN4, AGR, AGR1, EIR1, MM31, PIN2WAV6	Auxin efflux carrier family protein		−−	−−	AT1G70940^c^	T-DNA insertion line has serrated leaves	[77, 104]
			AT1G70940^c^						AT1G73590^a,b,c^	Mutants have fewer leaves with distorted shape	
			AT1G73590^a,b,c^								
			AT2G01420^c^								
			AT5G57090^c^								
Cell wall	*GRMZM2G445905*		AT5G44030^a^	CESA4, IRX5, NWS2	Cellulose synthase-like	−−	−−		AT5G44030^a^	T-DNA insertion line grows slower, plants are darker green and leaves are more narrow	[103]
Unknown	*GRMZM2G015295*	Adenosylhomo-cysteinase	AT3G23810^b,c^	SAHH2, EMB1395HOG1, MEE58, SAHH1,	S-adenosyl-l-homocysteine (SAH) hydrolase 2, S-adenosyl-L-homocysteine hydrolase		−−	−−	AT4G13940^a,b,c^	Loss of function mutants show increased leaf size, higher seed yields and delayed flowering, while overexpression plants show opposite phenotypes	[105]
			AT4G13940^a,b,c^								

*Arabidopsis* orthologs were determined using PLAZA3.0 web resource [147]. ^a^ Best hit family ortholog. ^b^ Tree-based ortholog. ^c^ Orthologous gene family. Double plus signs ("*++*") indicate transcript levels positively correlated with phenotypic trait (q_0.99_). Single plus signs ("*+*") indicate transcript levels positively correlated with phenotypic trait (q_random_). Double minus signs ("*−−*") indicate transcript levels negatively correlated with phenotypic trait (q_0.01_). *IAA* indole-3-acetic acid

In the intersection between LED_5-e_ and LL, we found genes related to entirely different processes than in the intersection between LER and LL, namely genes related to primary metabolism — amino acid synthesis and lipid metabolism — cell wall proteins, genes involved in cell vesicle transport, hormone signal transduction, subtilases, receptor kinases and stress-related genes. In addition, several transcription factors were identified that are commonly correlated with LL and LED_5-e_, belonging to the bHLH, HSF, NAC, SBP, SET and Trihelix families. Some examples of genes in the intersection of LL and LED_5-e_ (Table 4) are *GRMZM2G074267*, which shows homology to the auxin efflux carriers or PINs [77, 104], *GRMZM2G015295*, a S-adenosyl-L-homocysteine hydrolase showing homology to cytokinin binding protein HOG1 [105] and *GRMZM2G702026*, a homolog of *AUXIN RESPONSE FACTOR 1* (*ARF1*) [77].

LED_5-e_ and the other timing traits were positively correlated with “carbohydrate metabolism” (Fig. S6 in Additional file 1); accordingly, cluster 6 of the co-expression network, consisting predominantly of genes positively correlated with timing traits, was also enriched in this MapMan category (Fig. 4). The corresponding genes were mainly involved in starch biosynthesis. Availability of starch in leaves is a major determinant of plant growth since it provides a supply of carbon during the night, when no photosynthesis takes place but growth is nevertheless continuing [106, 107]. Our data suggest that RILs that have the potential to maintain high growth rates for longer periods of time have a modified balance between carbon supply and growth compared with other RILs. Tight regulation of starch biosynthesis is required to determine how much carbohydrate can be used during the day for growth and how much starch should be synthesized to provide the plant with carbon during the subsequent night [108]. The flux of carbon into starch is governed largely by regulation of the enzyme ADP-glucose pyrophosphorylase (AGPase). Transcript levels of one of the subunits of this enzyme [109, 110], GRMZM2G106213, showed a correlation in our dataset with the timing traits (Additional file 2), consistent with an increase in yield and biomass in several crops when altering AGPase activity [111–115]. Another example is a sucrose-phosphate synthase, GRMZM2G055331 (Additional file 2). Sucrose-phosphate synthase enzymes catalyze the rate limiting steps in the biosynthesis of sucrose and play an important role in carbon partitioning in the regulation of starch production versus sugar accumulation in many developmental processes [116]. In several species, increased or ectopic expression of sucrose-phosphate synthases resulted in an increase in plant size [117–119], while down-regulation resulted in a strong decrease in plant growth [120, 121].

Which cellular mechanism was affected in the mutant phenotype was not examined for the majority of the genes with a known role in leaf development, impeding further validation of a specific role of the (anti-)correlating genes in leaf growth rate or duration of leaf elongation. Our analysis, however, provides a potential framework to start deciphering the molecular networks underlying the trait LL and provides evidence that there are at least two mechanisms regulating leaf size.

### Leaf as an organ contributing to biomass

The shoot parameters FW and DW showed a strong correlation on the phenotypic level — a PCC of 0.893 (Table 2) — and this was also reflected by the high fraction of genes that (anti-)correlated with both traits: approximately two-thirds of the genes were commonly (anti-)correlated (Fig. 5e, f). In addition, seedling biomass showed a significant positive correlation with LER and Lwe, and to a lesser extent with LA, LL and DZ size, but not with Lwi, indicating that the majority of the final leaf size traits are important contributors to seedling biomass, next to leaf number and V-stage. The highest correlation was observed between seedling weight and LER and this was also observed in the PCA biplot (Fig. S4 in Additional file 1). This suggests that LER can be used as a proxy for seedling biomass. At the molecular level, 19 genes correlated with both LER and seedling biomass and 30 genes anti-correlated with both (Fig. 5e, f). These genes are involved in a variety of processes, such as cell organization, protein degradation and posttranslational modifications, transcriptional regulation (e.g., C_2_H_2_ zinc finger family protein, MYB domain protein and methyl-binding domain protein) and signaling. However, the numbers were too small to identify enriched processes.

Some genes in the intersections between LER and FW and/or DW have homologs in *Arabidopsis* for which perturbation mutants have phenotypes that hint at a role in shoot development (summarized in Table 6). For instance, *GRMZM2G091715* encodes an acyl carrier protein involved in de novo synthesis of fatty acids [122]; *GRMZM2G092595* shows homology to *FAB* genes encoding a phosphatidylinositol-3P 5-kinase important for endomembrane homeostasis [123]; Zm*CCD8*/*MAX4* (*GRMZM2G446858*) is a strigolactone biosynthetic gene [124]; *GRMZM2G149224* encodes a 3β-hydroxysteroid-dehydrogenase/decarboxylase required for plant sterol activation [125]; GRMZM2G170567 is a transcriptional activator [126]; GRMZM2G704093 is a homolog of *Arabidopsis* CUL4, part of an E3 ubiquitin ligase complex [127]. Also, for *Arabidopsis* homologs of *GRMZM2G406043*, *GRMZM2G346639* and *GRMZM2G094951* (*At3g04490*, *At5g53770* and *At2g37290*, respectively) a leaf or whole rosette phenotype was recently described in the corresponding T-DNA insertion mutants [77].

**Table 6:** Examples of genes for which expression levels are (anti-)correlated with fresh/dry weight and LER

MapMan	Gene	Maize description	*Arabidopsis* orthologs	*Arabidopsis* symbol	*Arabidopsis* description	LER	FW	DW	*Arabidopsis* orthologs with phenotype	Phenotype	Reference
Hormone metabolism	*GRMZM2G446858*	Carotenoid cleavage dioxygenase	AT4G32810^a,b,c^	CCD8, MAX4	Carotenoid cleavage dioxygenase 8	++	++	++	AT4G32810^a,b,c^	ZmCCD8/MAX4 is a stringolactone biosynthetic gene of which the knockout mutant shows a branching phenotype, comparable to the *Arabidopsis* orthologous mutant, and in addition shorter stature and smaller ears	[124, 155]
Protein degradation	*GRMZM2G704093*		AT5G46210^a,b^	CUL4	Cullin4	−−	−	−−	AT5G46210^a,b^	CUL4 is part of an E3 ubiquitin ligase complex. Loss of function mutants display reduced growth and aberrant leaf phenotypes	[127]
			At4G12100^b^								
			AT3G46910^b^								
Lipid metabolism	*GRMZM2G149224*	Sterol-4-alpha-carboxylate 3-dehydrogenase, decarboxylating	AT1G47290^b,c^	3βHSD/D1, AT3βHSD/D1, 3βHSD/D2, AT3βHSD/D2	3β-Hydroxysteroid-dehydrogenase/decarboxylase	−−	−−	−−	AT1G47290^b,c^	Overexpression of this 3β-hydroxysteroid dehydrogenase/decarboxylase required for plant sterol activation results in growth defects, such as shorter internodes	[125]
			AT2G26260^a,b,c^								
	*GRMZM2G091715*	Acyl carrier protein	AT4G25050^c^	ACP4, ACP1, ACP2, ACP3, ACP5	Acyl carrier protein	++	++	++	AT4G25050^c^	Reduced ACP4 levels result in a decreased lipid content and varying degrees of a bleached phenotype, smaller size and shorter bolts	[122]
			AT3G05020^a^								
			AT1G54580^c^								
			AT1G54630^c^								
			AT3G17790^c^								
Signaling	*GRMZM2G094951*		AT2G37290^c^		Ypt/Rab-GAP domain of gyp1p superfamily protein	−−	−−	−−	AT2G37290^c^	T-DNA line shows pale green leaves	[77]
			AT2G39280^c^								
			AT3G55020^a,b,c^								
	*GRMZM2G092595*		AT1G71010^a,b,c^	FAB1C, FAB1B, FAB1A	FORMS APLOID AND BINUCLEATE CELLS 1C, phosphatidylinositol-4-phosphate 5-kinase family protein, 1-phosphatidylinositol-4-phosphate 5-kinases, zinc ion binding, 1-phosphatidylinositol-3-phosphate 5-kinases	++	++	+	AT3G14270^c^	Loss of function and gain of function mutants display pleiotropic phenotypes primarily related to auxin signaling, including dwarfism and root growth inhibition	[123]
									AT4G33240^c^		
			AT3G14270^c^								
			AT4G33240^c^								
			AT1G34260^c^								
Development	*GRMZM2G170567*		AT5G18410^a,b^	KLK, PIR, PIR121, PIRP, SRA1	Transcription activators	−−	−	−−	AT5G18410^a,b^	Downregulation results in larger rosettes that are epinastic and paler green, next to additional developmental phenotypes	[126]
RNA processing	*GRMZM2G346639*		AT5G53770^a,b,c^		Nucleotidyl-transferase family protein	−−	−−	−−	AT5G53770^a,b,c^	T-DNA line shows smaller, more compact rosette with more roundish leaves	[77]
Unknown	*GRMZM2G406043*		AT3G04490^a,c^			−−	−−	−−	AT3G04490^a,c^	Smaller rosette size due to smaller leaves in T-DNA line	[77]

*Arabidopsis* orthologs determined using PLAZA3.0 web resource [147]. ^a^ Best hit family ortholog; ^b^ Tree-based ortholog; ^c^ Orthologous gene family. Double plus signs ("*++*") indicate transcript levels positively correlated with phenotypic trait (q_0.99_). Single plus signs ("*+*") indicate transcript levels positively correlated with phenotypic trait (q_random_). Double minus signs ("*−−*") indicate transcript levels negatively correlated with phenotypic trait (q_0.01_). Single minus signs ("*−*") indicate transcript levels negatively correlated with phenotypic trait (q_random_)

The positive correlations between final leaf size traits and seedling biomass indicated that these traits are important contributors to seedling biomass. Since LER showed the highest correlation with FW and DW, LER measurements that take place early in development might allow to identify subsets of plants in populations with high or low biomass. The identification of a number of genes in the intersection of LER and FW/DW for which a link with growth and development is shown further supports this hypothesis.

## Conclusions

In this study, we could successfully correlate variation in transcript levels in growing leaf tissue with variation for traits measured at later stages of development. This implies that dividing cells of a growing leaf already contain the molecular information underpinning the final phenotypes. Furthermore, we illustrate that breaking down complex traits such as leaf and seedling biomass into their components aids in determination of the most important contributors and their mutual association and facilitates the dissection of regulatory interactions. The relevance of our approach is also reflected by the presence of genes for which *Arabidopsis* homologs have a known function in leaf development or genetic perturbations display anticipated variation in leaf size in our gene sets correlating with the final leaf and shoot traits. Next to these known genes involved in leaf development, a large set of novel genes were also identified. In future studies, integration of phenotyping and transcriptomics data of additional mapping populations — since the mapping population used determines to a large extent the genetic variation that can be captured — and the combination with other forward genetic approaches, such as QTL and expression QTL analysis, will allow for selection of putative regulators of leaf growth that can be used for further analysis in genetic modification approaches or as biomarkers for leaf size traits.

## Materials and methods

### Genetic material

The RIL population used in this study is derived from a cross between parental lines B73 and H99, followed by 12 generations of self-pollination. In total, 142 RILs were generated and 223 markers were used for mapping these RILs [40]. A randomly chosen subset of 103 RILs was analyzed in this study.

### Growth conditions, measured traits and sampling

All traits were measured in a series of experiments in a single growth chamber. RILs were grown in a randomized design each time along with their respective parents. Experiments were conducted under controlled growth chamber conditions (24 °C, 55 % relative humidity, light intensity of 170 mmol m^−2^ s^−1^ photosynthetic active radiation, in a 16 h/8 h day–night cycle). Since the focus of our research was on leaf development, primarily leaf size traits were determined. The traits measured for leaf 4 were: final leaf 4 area (LA), final leaf 4 width (Lwi), final leaf 4 weight (Lwe), final leaf 4 blade weight, final leaf 4 length (LL), leaf 4 elongation rate (LER), DZ size of leaf 4 at steady state growth, time point of leaf 4 emergence, time point of maximal LER (T_m_), time point when leaf 4 reaches its final length (T_e_) and leaf elongation duration (LED_5-e_). Since results for Lwe and leaf 4 blade weight were highly correlated, only results for Lwe are shown. LER and DZ size were determined as described previously by Rymen et al*.* [41]. Briefly, LER was determined by measuring the leaf length, using the soil level as a reference point, on a daily basis from the time of emergence of leaf 4 until the leaf was fully grown and calculating the average growth rate during the steady state growth phase. DZ size was estimated as the distance between the base of the leaf and the most distal mitotic cell in the epidermis that could be visualized after staining with 4′,6-diamidino-2-phenyindole (DAPI). T_m_, T_e_ and LED_5-e_ were determined as described before [42]. Additionally, at a fixed time point after sowing (after 27 days), fresh weight, dry weight, V-stage and total number of leaves of the whole seedling were determined. V-stage and total number of leaves were not determined for all RILs, but for a selection of 42. All traits were determined for six plants per RIL, except for DZ size (three plants per RIL) and time point of leaf 4 emergence (19 plants per RIL). Simultaneously with phenotyping, plants were sampled for RNA sequencing. Since we preferred to grow plants for phenotypic analysis, including determination of DZ size, and RNA sequencing simultaneously, it was not feasible to sample the total DZ. Therefore, we sampled the first 0.5 cm of the most basal part of leaf 4 three days after appearance, always at the same time of the day to minimize diurnal effects. This zone of the leaf is at that stage fully proliferative for all RILs we examined. For each parent we had three biological and three technical replicates, each pool consisting of proliferative tissue of four plants. For the RILs, one biological replicate, consisting of proliferative tissue of four plants, was sampled for RNA sequencing. Total RNA was extracted using Trizol (Invitrogen) according to the manufacturer’s instructions, followed by DNA digestion using the RNase-free DNase I kit (Qiagen).

### Data analysis

#### PCCs and PCA analysis of phenotypic data

Pearson correlations among the traits were calculated on the means of the RILs and two parental lines in SPSS (SPSS Inc., Chicago, IL, USA). PCA was performed as a dimensionality reduction technique on the centered and scaled phenotype data, using the prcomp function in R.

#### RNA sequencing analysis

Library preparation was done using the TruSeq RNA Sample Preparation Kit v2 (Illumina). In brief, poly(A)-containing mRNA molecules were reverse transcribed, double-stranded cDNA was generated and adapters ligated. After quality control using a 2100 Bioanalyzer (Agilent), clusters were generated through amplification using the TruSeq PE Cluster Kit v3-cBot-HS kit (Illumina) followed by sequencing on an Illumina HiSeq2000 the TruSeq SBS Kit v3-HS (Illumina). Sequencing was performed in paired-end mode with a read length of 100 bp.

The quality of the raw data was verified with FastQC [128] (version 0.9.1). Next, quality filtering was performed using the FASTX-Toolkit [129] (version 0.0.13): reads were globally filtered so that, for at least 75% of the reads, the quality exceeds Q10 and 3’ trimming was performed to remove bases with a quality below Q20, ensuring a minimum length of 35 bp remaining. Re-pairing was performed using a custom perl script. Reads were subsequently mapped to the maize B73 reference genome (5b) using GSNAP [130] allowing maximally five mismatches. The concordantly paired reads that uniquely map to the genome were used for quantification on the gene level with htseq-count from the HTSeq.py python package [131].

It has been reported that inbred lines of maize are very divergent [132]. This could introduce artifacts in the mapping of reads and therefore inaccurate transcript quantification. Therefore, we selected for genes that are conserved between inbred lines. To make this selection more robust, we included eight inbred lines in this selection procedure, among which were the two parental lines of the RIL population studied here. RNA-seq data of proliferative tissue for these eight inbred lines (M.E. Pè, personal communication) was mapped to the B73 reference genome. A coverage cutoff was applied, using the R/Bioconductor package with default HTSFilter parameter settings [133]. This coverage cutoff retained 50 % of the genes (19,948) which are expressed in at least one of the parents.

Next, SNP calling was performed. The reads of the different libraries were preprocessed separately. Read sorting was done using SAMtools version 0.1.18 [134] and deduplication using Picard MarkDuplicates version 1.56 [135]. Subsequently, variants were called using GATK version 2.5.2 [136]. First, all read libraries were recalibrated using the tool BaseRecalibrator. A high quality SNP set was used as so-called known sites and this set was generated by UnifiedGenotyper using a quality threshold of 50. Next, an 18-way variant calling was performed using UnifiedGenotyper. Three variant sets were generated: a raw variant set by setting a quality threshold of 30, and a high quality SNP and INDEL set by setting a quality threshold of 50. Quality scores of the raw variants were recalibrated using the high quality variant sets and the tools VariantRecalibrator and ApplyRecalibration. For SNPs, we set the VariantRecalibrator options maxGaussians to 10, percentBad to 0.01, minNumBad to 1000 and an to QD, MQRankSum, ReadPosRankSum, FS and DP. For INDELs, we set the VariantRecalibrator options maxGaussians to 4, percentBad to 0.05, minNumBad to 2500 and an to mQRankSum, ReadPosRankSum, FS and DP. In both cases, we set the ApplyRecalibration option ts_filter_level to 99.0. In case of INDELs, the recalibrated SNP set was used. Using BEDops version 2.2.0 [137], BEDTools version 2.16.1 [138] and VCFtools version 0.1.10 [139], we selected only variants present in the exon regions as defined in the ZmB73_5b filtered gene set GFF file [140]. Finally, genes with no more than 1.75 % of SNPs were selected. By applying this threshold, 75 % of the expressed genes were retained. This resulted in a set of 15,051 selected genes.

#### Normalization and transformation of RNA-seq count data

Count data of the filtered set of 15,051 transcripts were normalized for library size with the default normalization methods in the DESeq2 package version 1.2.10 [141] in R version 3.0.2 [142]. Transcripts expressed in less than 5 % of samples (transcript count > 0) were removed. An inverse hyperbolic sine transformation was applied on the remaining transcript levels ("asinh" function in R), which is able to transform the zero counts. Additionally, the 5 % least varying transcripts (based on the coefficient of variation) were removed from further analyses.

#### Correlation analysis between phenotype and transcriptome

PCCs were calculated between each transcript and all traits over all RILs and parents. For each trait, the q_0.01_ and q_0.99_ quantiles of the set of transcript–trait PCC values were calculated. The genes with PCC higher and lower than the q_0.99_ and q_0.01_, respectively, showed no bias in absolute expression levels or expression range between RILs compared with the filtered gene set of 15,051 genes. In order to compare the general linear correlation tendency of transcripts and traits, a distribution of correlation coefficients expected by chance was calculated by permuting the trait values 1000 times and calculating the q_0.01_ and q_0.99_ quantiles for each permutation. The mean q_0.01_ and q_0.99_ quantiles were taken as the reference correlation coefficients expected by chance across the 105 parent and RIL samples. For the majority of the analyses performed, we focused on the genes with PCC lower and higher than the q_0.01_ and q_0.99_ quantiles, respectively, of the set of transcript–trait PCC values, indicated as the (anti-)correlating gene sets. Few analyses were based on the gene sets with PCC higher than expected by chance, indicated as q_random_ correlating gene sets. The traits LN and V-stage were not analyzed using this approach since the phenotyping data were only available for 42 of the RILs.

The expression patterns of the (anti-)correlating sets of genes in the different RILs and parental lines were visualized in MeV [143]. Data were adjusted by normalizing the genes/row and color scale limits were set at −3 and 3 as the lower limit and upper limit, respectively, since these numbers approached the minimal and maximal data values after normalization. For many traits, there are particular RILs for which the expression levels of all correlating and anti-correlating genes are higher and lower, respectively, than for other RILs, which becomes visually apparent as green and red ribbons in Fig. 2 and Fig. S1 in Additional file 1. This is most likely not due to normalization errors since the RILs showing these brighter green and red ribbons differ from trait to trait (Fig. S5 in Additional file 1).

#### Correlation network

A transcript expression correlation network was calculated across all transcripts correlated with at least one trait, with transcripts as nodes and Pearson correlation values between transcripts as edge weights. For clustering and visualization, edges with correlation values below 0.6 were discarded. The resulting weighted network was clustered with the Markov cluster algorithm (MCL) [55] using clusterMaker version 1.10 [144] with granularity (inflation) parameter 2 and default advanced parameters, and visualized in Cytoscape version 3.1.0. A circular layout was used for clusters consisting of at least 15 transcripts; the rest of the network was visualized with the prefuse force directed layout.

#### Functional enrichment analysis

Functional enrichment analyses of the transcript clusters were based on the pathway annotations defined in MapMan [54]. The file with mapped maize transcripts and pathways was downloaded from the MapMan webpage [145]. For enrichment analyses of MapMan categories, the GOseq package [146] was used in R. The function is a priori programmed for gene ontology enrichment, but it allows for mapping user-defined categories. Additionally, it allows for analyses that incorporate transcript lengths. As a transcript background, the filtered list of 15,051 transcripts was used and an uncorrected *p* value of 0.01 was used as cutoff for selection. Biological interpretation was focused on MapMan categories that contained at least five genes. Functional annotation, mostly transferred from model species such as *Arabidopsis*, was determined using the online resource PLAZA3.0 [147].

Transcriptome data from this article have been submitted to the ArrayExpress data libraries (eight parental lines E-MTAB-3173; RILs E-MTAB-3758). Phenotyping data are available in Additional file 3.

## Additional files

Additional file 1: Figure S1. Mean minimum–maximum normalized values for all analyzed traits in parental lines and RILs. **Fig. S2** Some examples of RILs and the parental lines at seedling stage, 27 days after sowing. **Fig. S3** Coefficient of variation for the different traits measured. **Fig. S4** PCA analysis of the phenotype data of the population. **Fig. S5** Expression patterns of the top 1 % of genes (anti-)correlated with the different traits. **Fig. S6** Overrepresented MapMan categories of top 1 % of genes (anti-)correlated with phenotypic traits. **Fig. S7** Overrepresented MapMan categories of top 1 % of genes (anti-)correlated with final leaf size traits. (PDF 1976 kb) Additional file 2: Table S1. List of 1740 genes with expression levels in the leaf division zone of the RILs (anti-)correlating with at least one of the traits. (XLSX 363 kb) Additional file 3: Table S2. All phenotyping data per plant and averages per line. (XLSX 188 kb)

## Abbreviations

bp: base pair
DW: dry weight
DZ: division zone
FW: fresh weight
LA: leaf area
LED_5-e_: leaf elongation duration from a leaf of 5 mm until final length
LER: leaf elongation rate
LL: leaf length
LN: leaf number
Lwe: leaf weight
Lwi: leaf width
PCA: principal component analysis
PCC: Pearson correlation coefficient
QTL: quantitative trait loci
RIL: recombinant inbred line
SNP: single nucleotide polymorphism
T_e_: time between sowing and reaching final length
T_m_: time between sowing and reaching maximal growth rate
