# Supplementary material for: Correlation analysis of the transcriptome of growing leaves with mature leaf parameters in a maize RIL population
Source: Genome Biol. 2015 Sep 11;16(1):168. doi: 10.1186/s13059-015-0735-9 (PMC4566308; doi:10.1186/s13059-015-0735-9)
Supplement: Additional file 1: Figure S1. — Mean minimum–maximum normalized values for all analyzed traits in parental lines and RILs. Fig. S2 Some examples of RILs and the parental lines at seedling stage, 27 days after sowing. Fig. S3 Coefficient of variation for the different traits measured. Fig. S4 PCA analysis of the phenotype data of the population. Fig. S5 Expression patterns of the top 1 % of genes (anti-)correlated with the different traits. Fig. S6 Overrepresented MapMan categories of top 1 % of genes (anti-)correlated with phenotypic traits. Fig. S7 Overrepresented MapMan categories of top 1 % of genes (anti-)correlated with final leaf size traits. (PDF 1976 kb) [file 13059_2015_735_MOESM1_ESM.pdf]

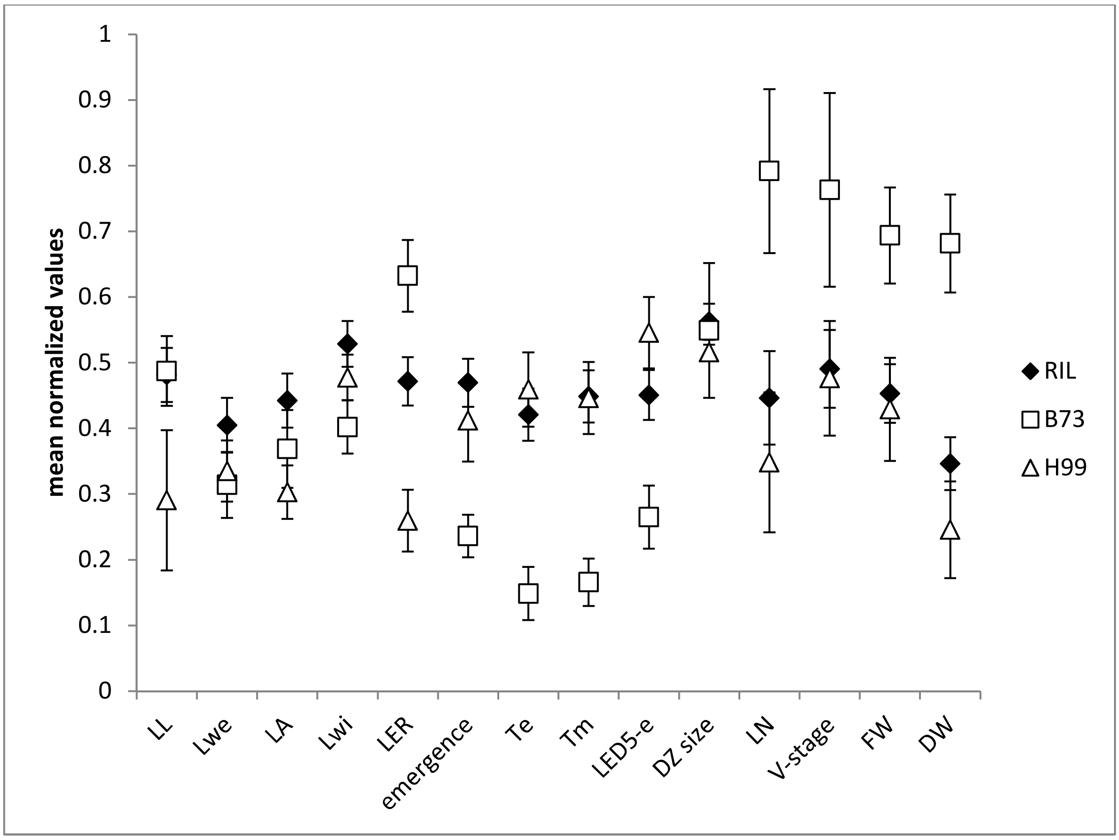

Figure S1 Mean minimum-maximum normalized values for all analyzed traits in parental lines and RILs. Error bars indicate 95% confidence interval. LL: leaf 4 final length, Lwe: leaf 4 final weight, LA: leaf 4 final area, Lwi: leaf 4 final width, LER: leaf 4 elongation rate, T_m: time to maximal LER, T_e: time to final leaf length, LED: leaf elongation duration, DZ size: leaf 4 division zone size, LN: leaf number 27 days after sowing, FW: fresh weight 27 days after sowing, DW: dry weight 27 days after sowing.

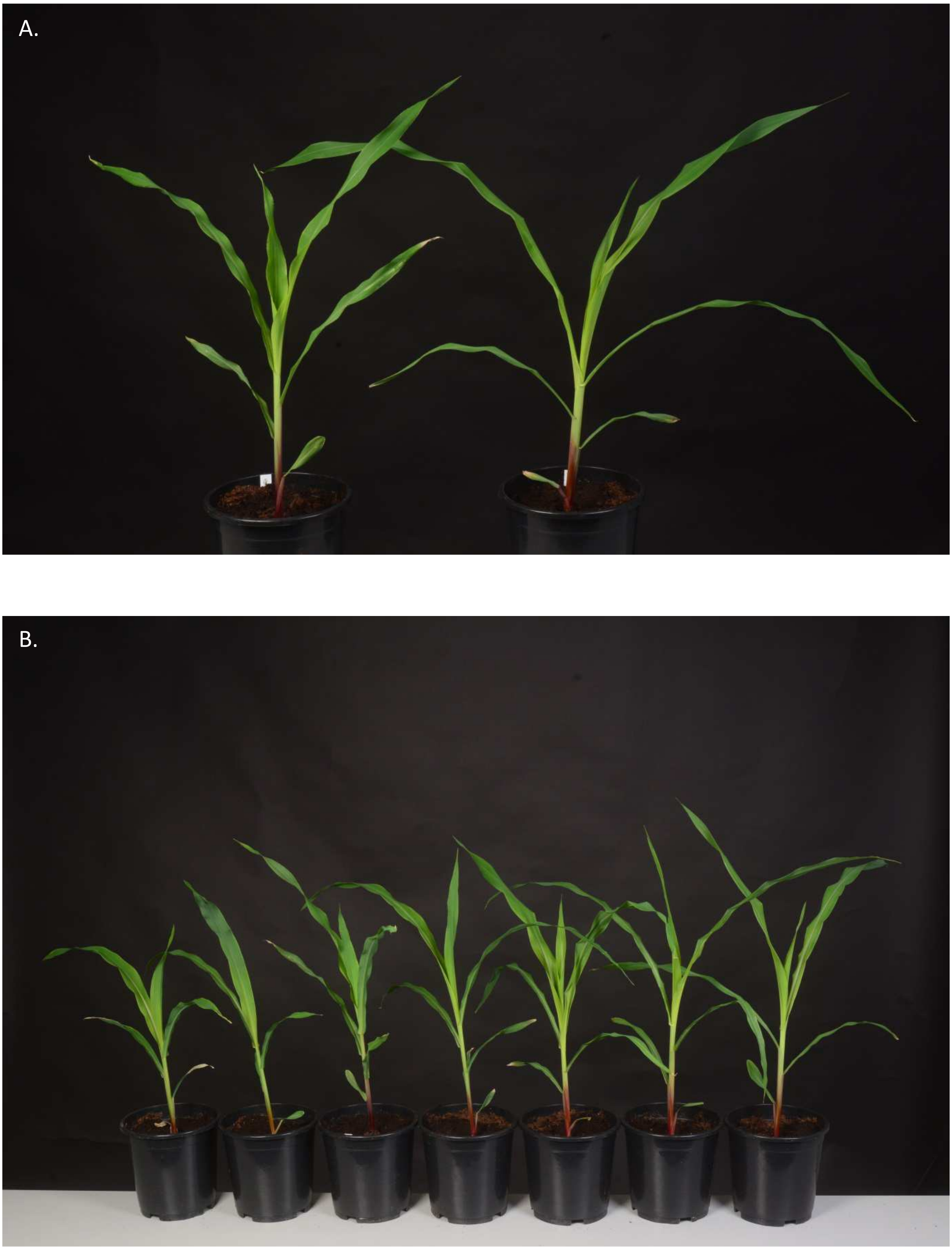

Figure S2 Some examples of RILs and the parental lines at seedling stage, 27 days after sowing. A. H99 (left) and B73 (right); B. From left to right: RIL100, RIL51, H99, RIL61, B73, RIL113, RIL89.

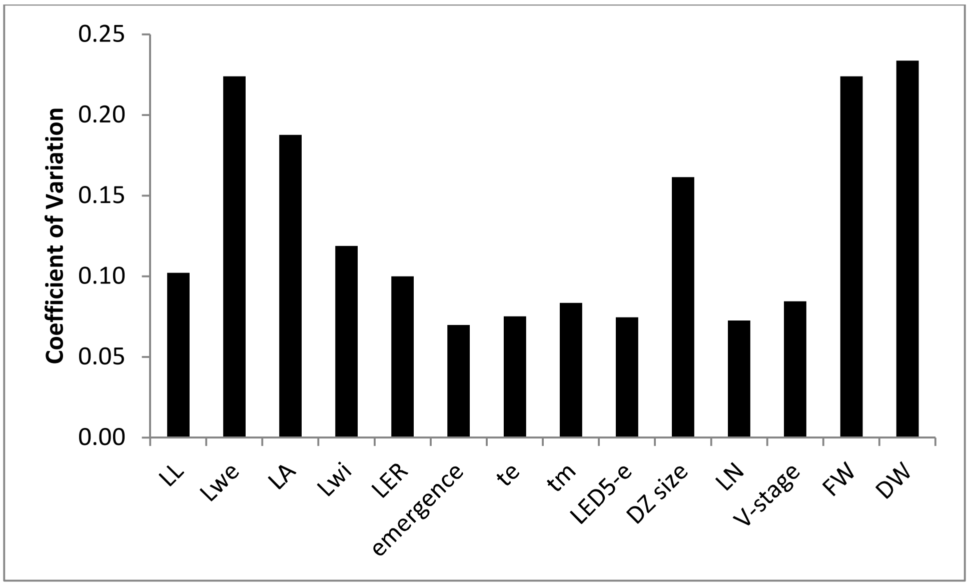

Figure S3 Coefficient of variation for the different traits measured.
LL: leaf 4 final length, Lwe: leaf 4 final weight, LA: leaf 4 final area, Lwi: leaf 4 final width, LER: leaf 4 elongation rate, T_m: time to maximal LER, T_e: time to final leaf length, LED_{5-e}: leaf elongation duration, DZS: leaf 4 division zone size, LN: leaf number, FW: fresh weight 27 days after sowing, DW: dry weight 27 days after sowing.

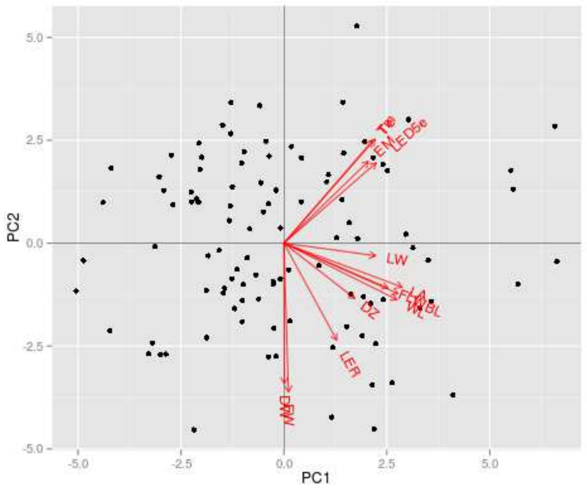

Figure S4 PCA analysis of the phenotype data of the population. Shown is a biplot of the first two principal components of the phenotype data. Black dots correspond to the different samples, while the red arrows correspond to the different phenotypes. LL: leaf 4 final length, Lwe: leaf 4 final weight, LA: leaf 4 final area, Lwi: leaf 4 final width, LER: leaf 4 elongation rate, T_m: time to maximal LER, T_e: time to final leaf length, $LED_{5\text{-}e}$: leaf elongation duration, DZS: leaf 4 division zone size, FW: fresh weight 27 days after sowing, DW: dry weight 27 days after sowing.

Leaf weight

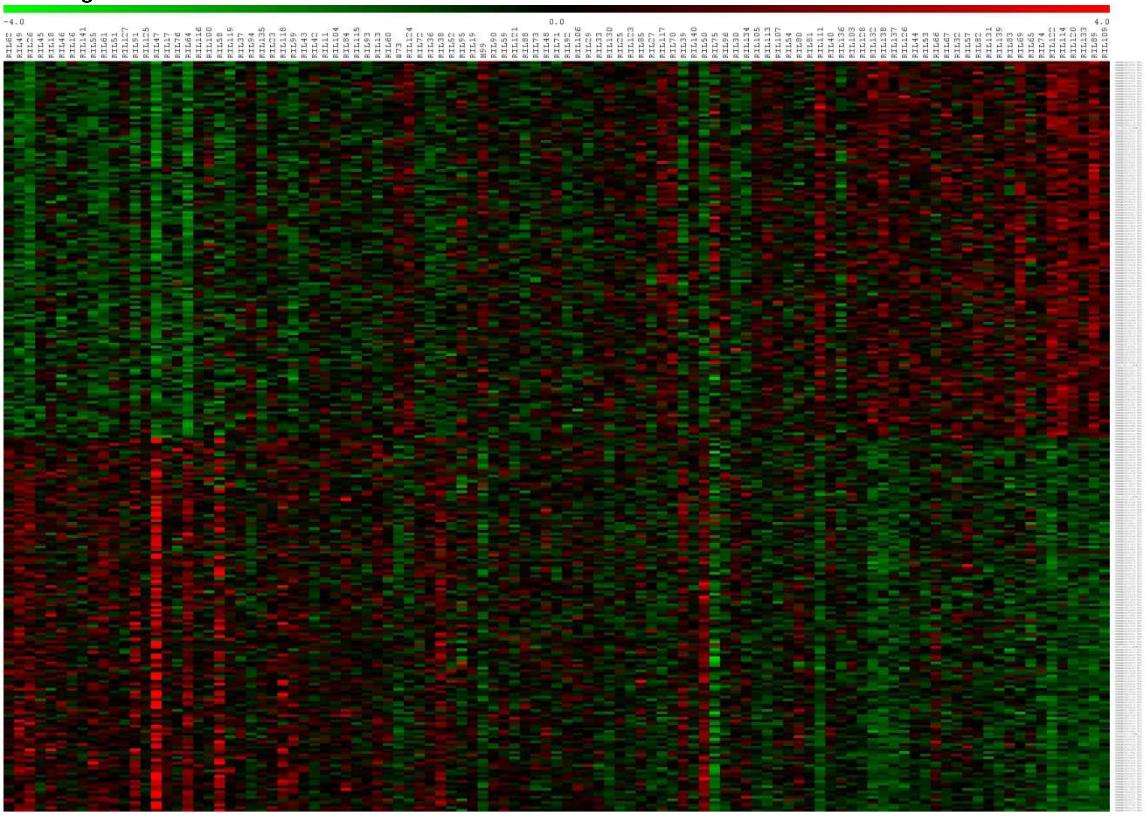

Leaf area

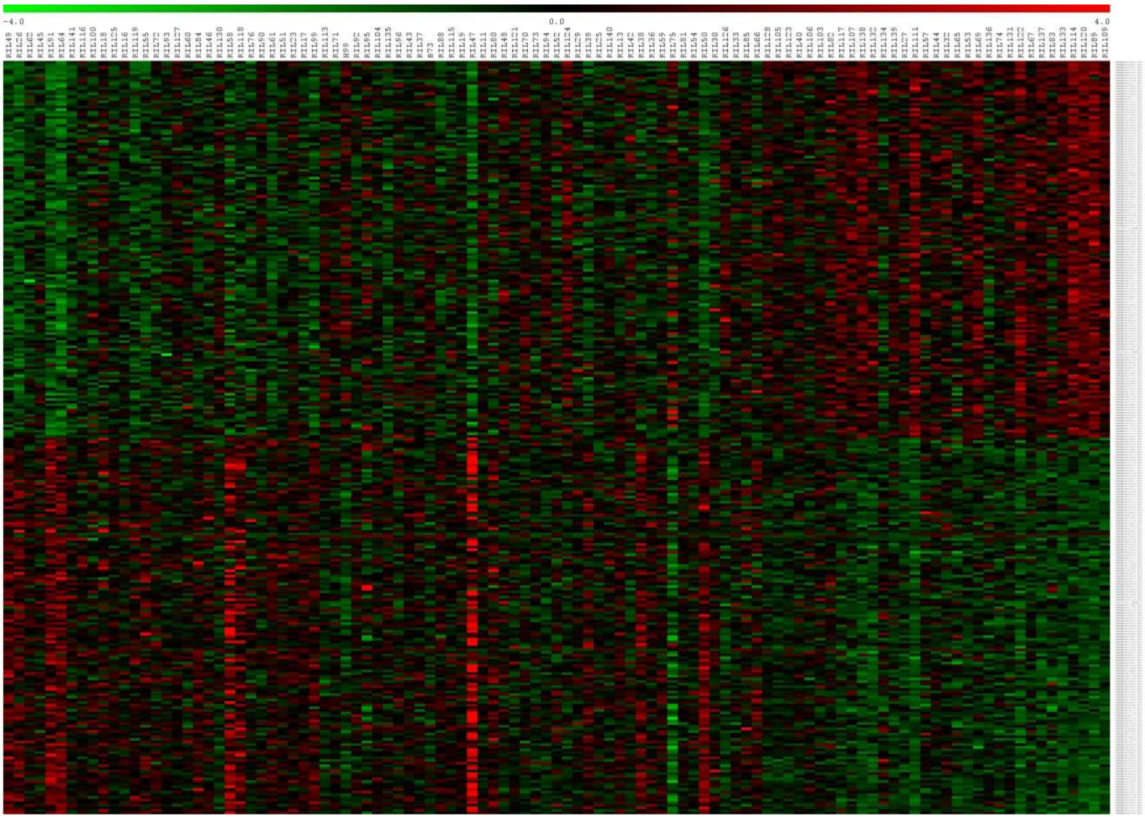

Leaf width

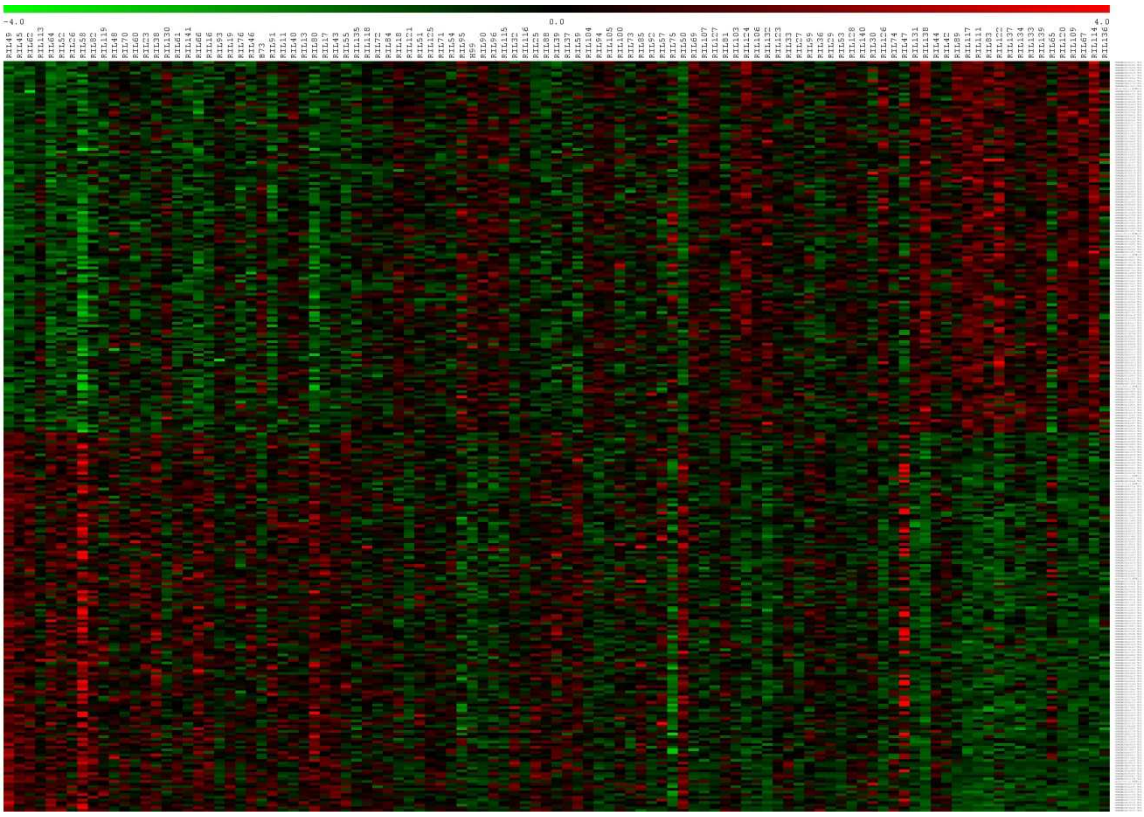

Leaf elongation rate

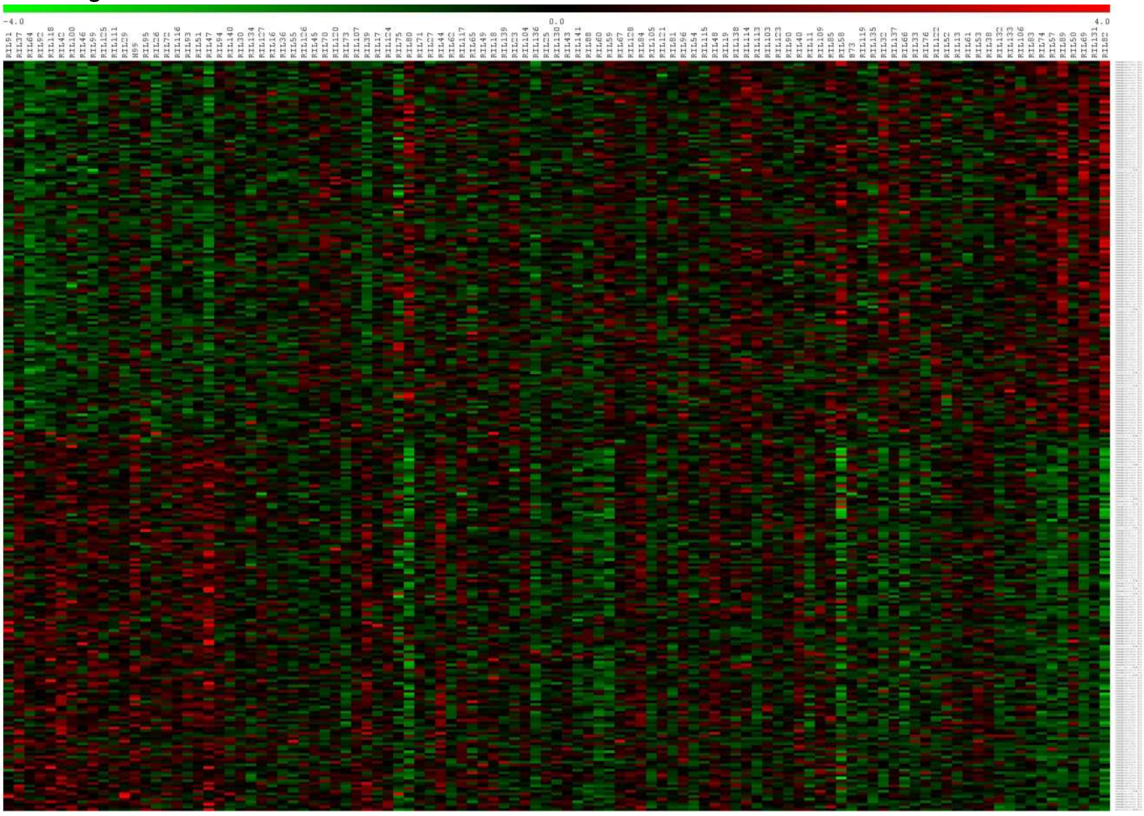

emergence

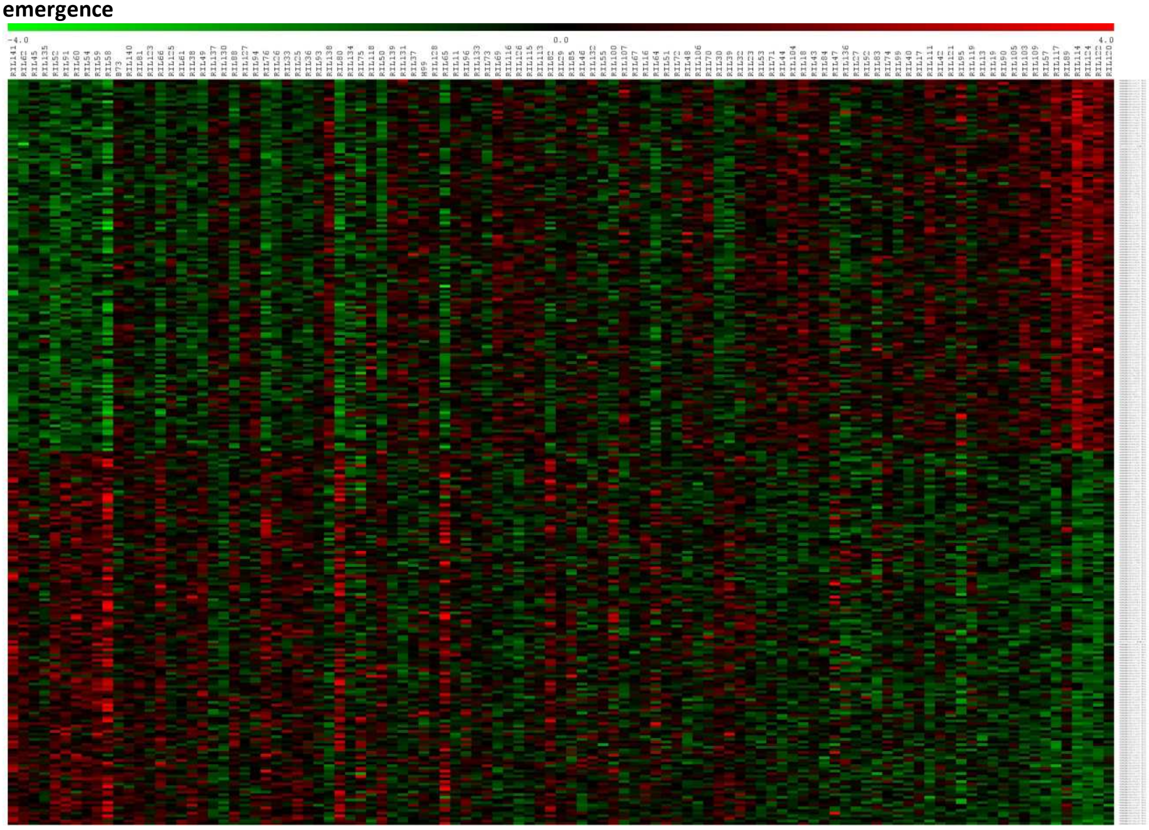

T_m

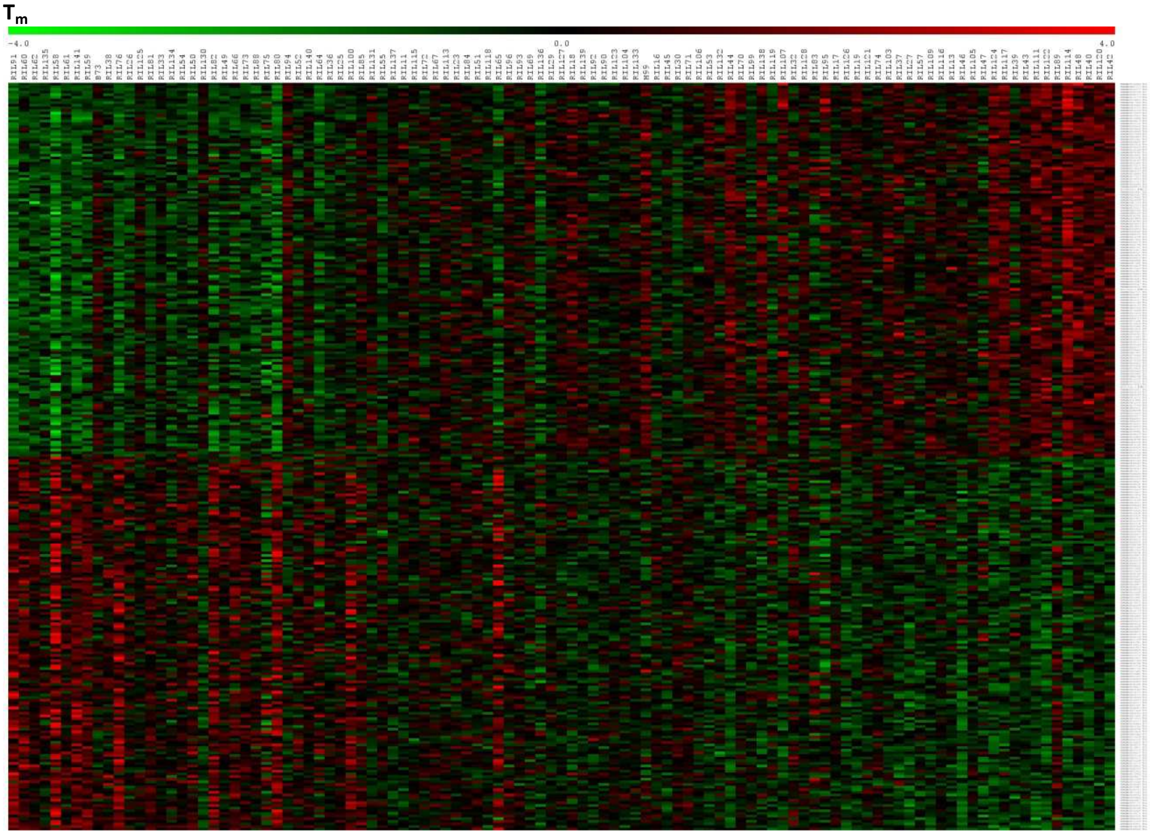

T_e

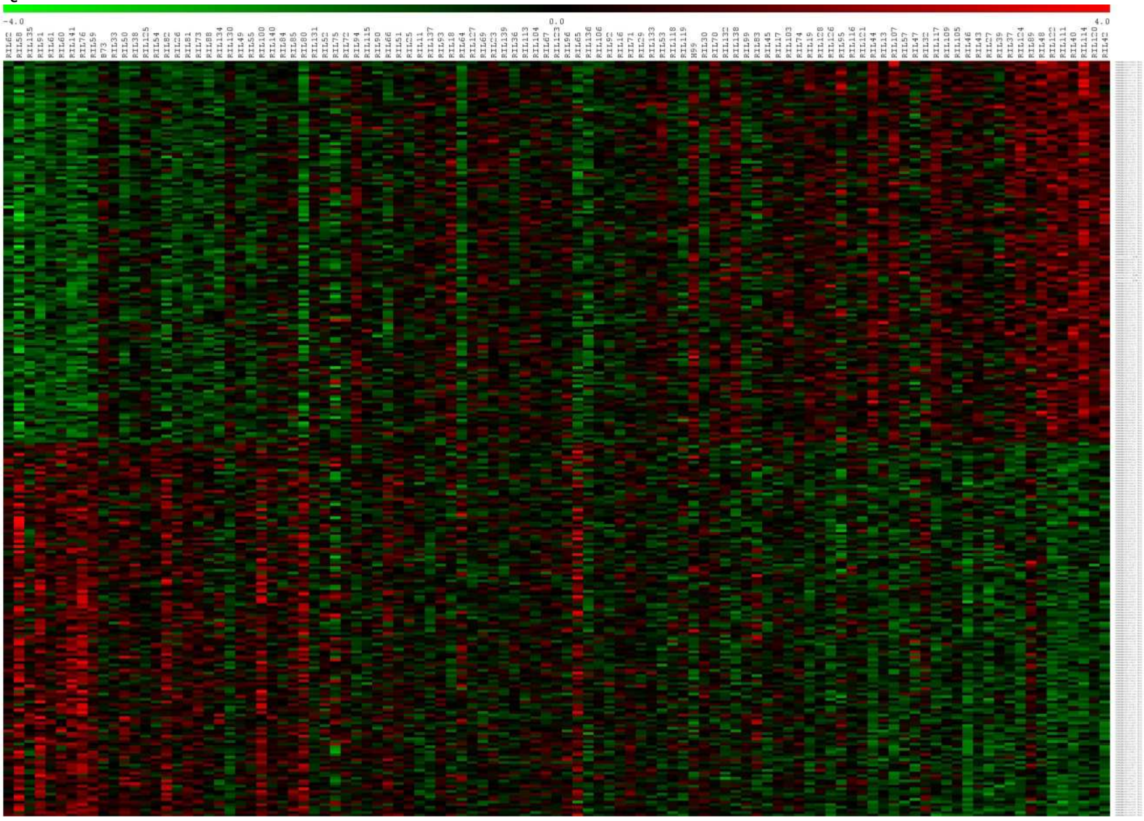

$LED_{5\text{-}e}$

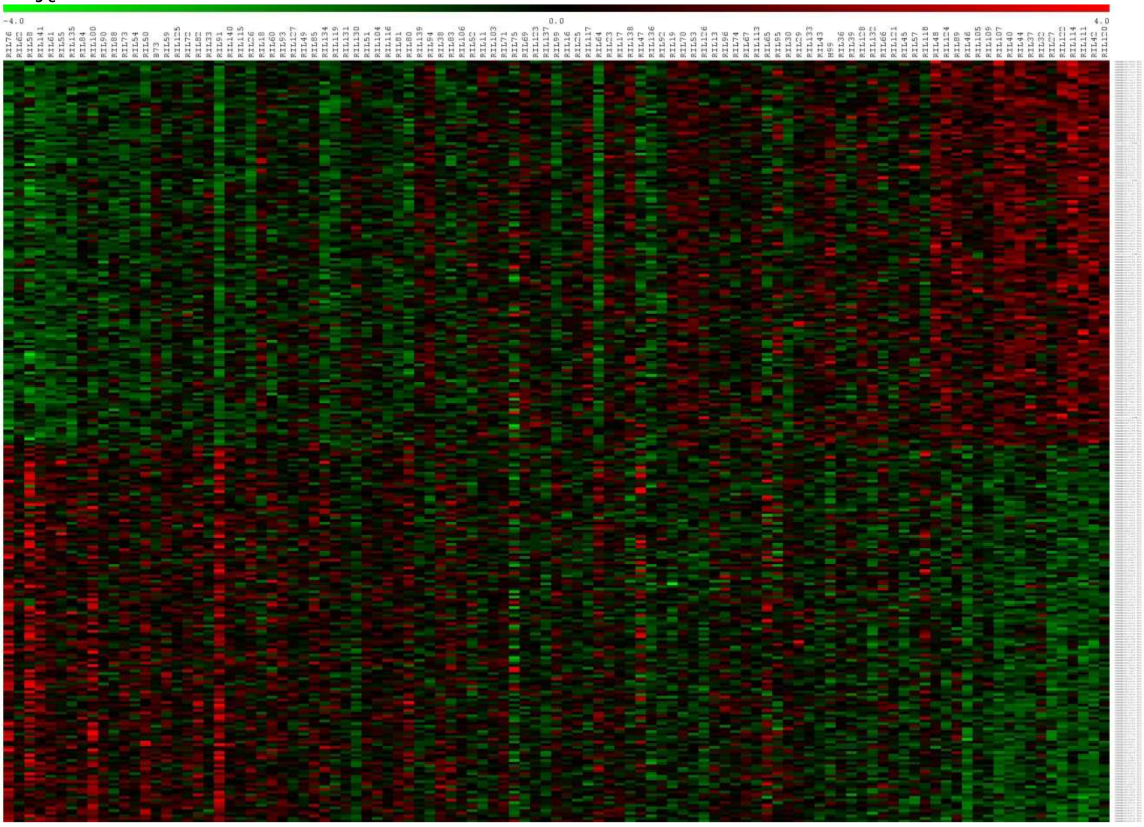

Division zone size

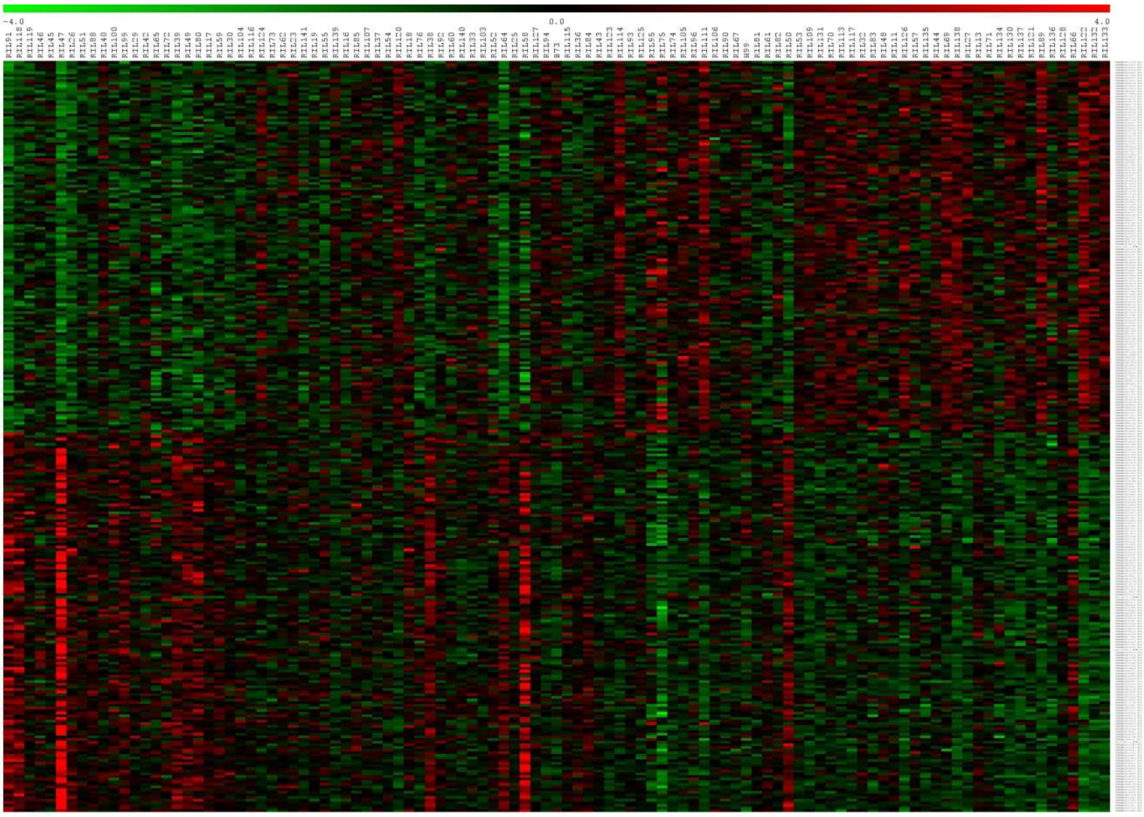

Fresh weight

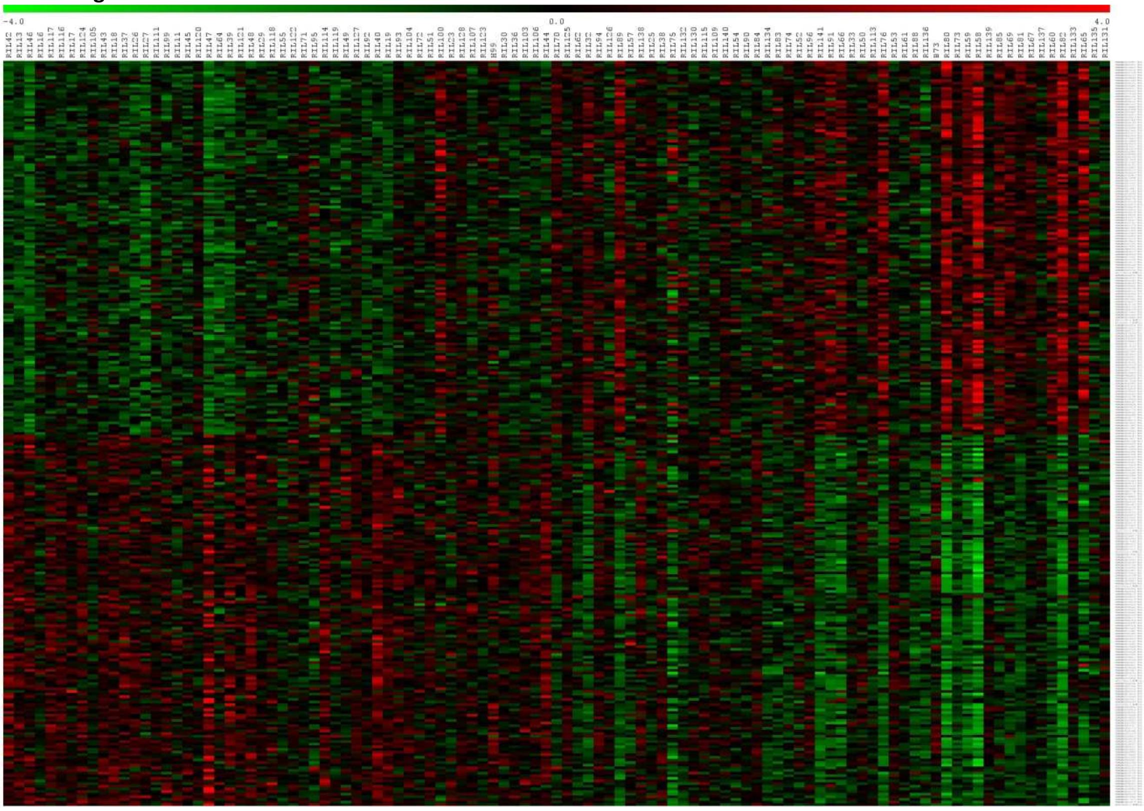

Dry weight

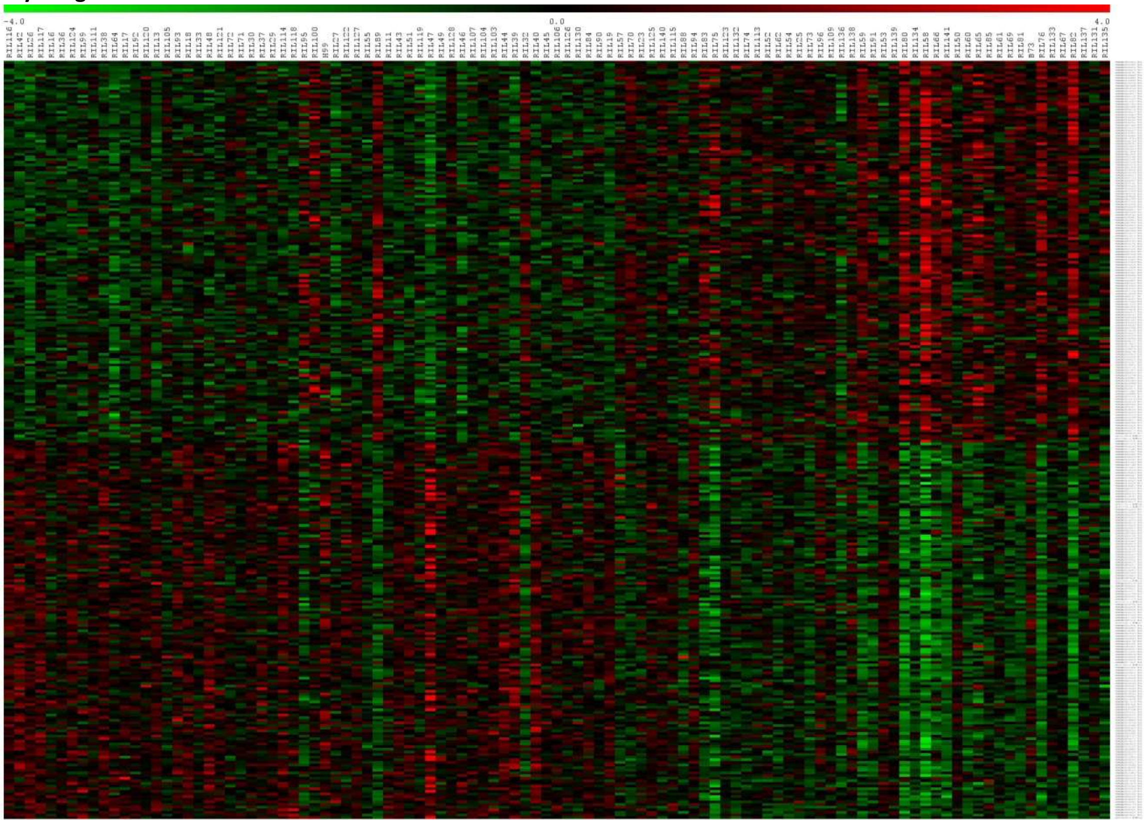

Figure S5 Expression patterns of the top one percent genes (anti-)correlated with the different traits. Columns represent the 103 RILs and parental lines B73 and H99 organized from small (left) to large (right) for the specific trait. Genes, shown in the rows, above the line are correlating genes, below the line anti-correlating genes. Green: low expression, red: high expression.

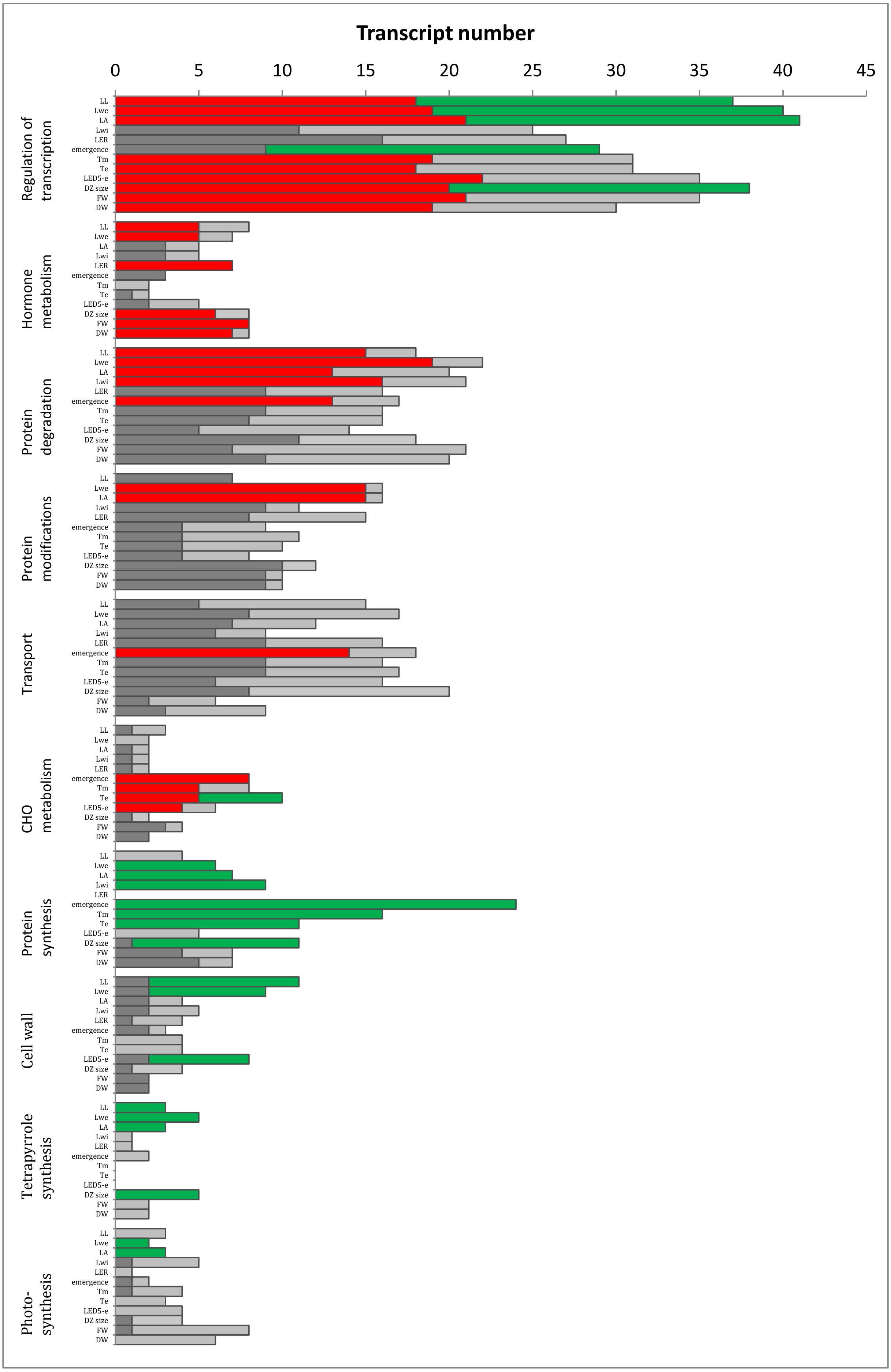

Transcript number
0
5
10
15
20
25
30
35
40
45
Regulation of transcription
Hormone metabolism
Protein degradation
Protein modifications
Transport
CHO metabolism
Protein synthesis
Cell wall
Tetrapyrrole synthesis
Photo-synthesis
LL
Lwe
LA
Lwi
LER
emergence
Tm
Te
LED5-e
DZ size
FW
DW

Figure S6 Overrepresented MapMan categories of top one percent genes (anti-)correlated with phenotypic traits. Positive correlation in dark grey bars, negative correlation in light grey bars. Red (positive correlation) and green bars (negative correlation) indicate enrichment for the functional category using Fisher's exact test (P<0.05). LL: leaf 4 final length, Lwe: leaf 4 final weight, LA: leaf 4 final area, Lwi: leaf 4 final width, LER: leaf 4 elongation rate, T_m: time to maximal LER, T_e: time to final leaf length, LED: leaf elongation duration, DZ size: leaf 4 division zone size, FW: fresh weight 27 days after sowing, DW: dry weight 27 days after sowing.

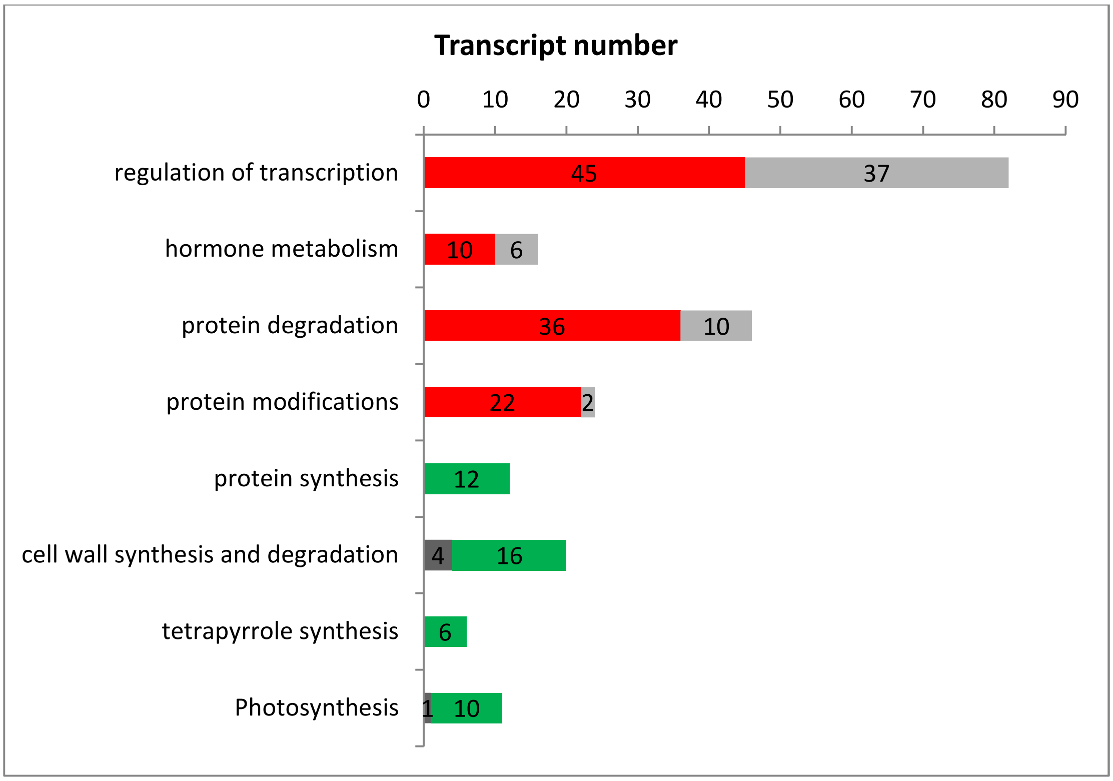

Figure S7 Overrepresented MapMan categories of top one percent genes (anti-)correlated with final leaf size traits. Positive correlation in dark grey bars, negative correlation in light grey bars. Red (positive correlation) and green bars (negative correlation) indicate enrichment for the functional category using Fisher's exact test (P<0.05).
